# Selective depletion of HBV-infected hepatocytes by class A capsid assembly modulators requires high levels of intrahepatic HBV core protein

**DOI:** 10.1128/aac.00420-24

**Published:** 2024-05-23

**Authors:** Dmytro Kornyeyev, Zhijuan Song, Stacey Eng, Cameron Soulette, Ricardo Ramirez, Jennifer Tang, Qin Yue, Raju Subramanian, Shiva Zaboli, Christina Moon, Jane Tam, Jens Brodbeck, Abhishek Aggarwal, Lauri Diehl, Simon P. Fletcher, Anastasia Hyrina, Meghan M. Holdorf, Dara Burdette

**Affiliations:** 1 Gilead Sciences, Inc., Foster City, California, USA; IrsiCaixa Institut de Recerca de la Sida, Barcelona, Spain

**Keywords:** capsid assembly modulator, hepatitis B virus

## Abstract

Capsid assembly mediated by hepatitis B virus (HBV) core protein (HBc) is an essential part of the HBV replication cycle, which is the target for different classes of capsid assembly modulators (CAMs). While both CAM-A (“aberrant”) and CAM-E (“empty”) disrupt nucleocapsid assembly and reduce extracellular HBV DNA, CAM-As can also reduce extracellular HBV surface antigen (HBsAg) by triggering apoptosis of HBV-infected cells in preclinical mouse models. However, there have not been substantial HBsAg declines in chronic hepatitis B (CHB) patients treated with CAM-As to date. To investigate this disconnect, we characterized the antiviral activity of tool CAM compounds in HBV-infected primary human hepatocytes (PHHs), as well as in HBV-infected human liver chimeric mice and mice transduced with adeno-associated virus-HBV. Mechanistic studies in HBV-infected PHH revealed that CAM-A, but not CAM-E, induced a dose-dependent aggregation of HBc in the nucleus which is negatively regulated by the ubiquitin-binding protein p62. We confirmed that CAM-A, but not CAM-E, induced HBc-positive cell death in both mouse models via induction of apoptotic and inflammatory pathways and demonstrated that the degree of HBV-positive cell loss was positively correlated with intrahepatic HBc levels. Importantly, we determined that there is a significantly lower level of HBc per hepatocyte in CHB patient liver biopsies than in either of the HBV mouse models. Taken together, these data confirm that CAM-As have a unique secondary mechanism with the potential to kill HBc-positive hepatocytes. However, this secondary mechanism appears to require higher intrahepatic HBc levels than is typically observed in CHB patients, thereby limiting the therapeutic potential.

## INTRODUCTION

Hepatitis B virus (HBV) is a 3.2 kb partially double-stranded hepatotropic DNA virus that replicates through reverse transcription of an RNA intermediate. Persistence of HBV is attributed to, at least in part, the stable nuclear pool of covalently closed circular DNA (cccDNA) which serves as the transcriptional template for all viral transcripts and proteins required for the viral life cycle, including hepatitis B surface antigen (HBsAg). In addition to cccDNA, integration of HBV into the host genome may also contribute to persistence as it is a major source of HBsAg (1, 2). Chronic hepatitis B (CHB) is a global healthcare concern as patients are at increased risk of liver diseases including cirrhosis and hepatocellular carcinoma (3). The current standard of care for CHB is typically life-long treatment with nucleos(t)ide analogs (NAs), which suppress viral replication but do not directly reduce the levels of cccDNA or integrated HBV. Therefore, they rarely lead to a cure (4–7). Therefore, treatments focused on the elimination of hepatocytes containing cccDNA and integrated HBV are needed.

Capsid assembly modulators (CAMs) are being explored as a new treatment option for CHB. CAMs target HBV core protein (HBc) at the dimer interface disrupting the assembly of the viral capsid, which ultimately leads to a reduced production of infectious virions. Various HBV CAMs have been developed and are classified under two major types based on the resulting phenotype of the capsid (8). Molecules belonging to class A CAMs (CAM-A), e.g., heteroaryldihydropyrimidine (HAP), misdirect capsid assembly producing an aberrant core structure unable to encapsulate pgRNA (9, 10). Class E CAMs (CAM-E), e.g., sulfamoylbenzamide and phenylpropenamide derivatives, promote the production of regularly shaped empty capsids (11–13). Both classes of CAMs reduce extracellular HBV DNA and RNA. However, in contrast to CAM-E compounds, the CAM-As (RG7907, ALG-005398, and GLS-4) demonstrate nuclear HBc aggregation leading ultimately to a loss of cell viability resulting in cell death in HBc-positive cell lines (14–16). Of note, this CAM-A-induced cell death has not been observed to date in HBV-infected primary human hepatocytes (PHH). Cell death of HBc-positive cells resulting in the reduction of HBsAg is also observed across different preclinical mouse models (14–17). Nevertheless, clinical studies of RG7907 in combination with an NA demonstrated a reduction in serum HBV DNA and HBV RNA below the lower limit of quantification in both treatment-naïve and NA-suppressed patients. However, no significant reduction of HBsAg in either treatment group was observed (18, 19). Similar results were demonstrated with GLS4, in which only a modest HBsAg reduction (0.4 log_10_ IU/mL) in treatment-naïve hepatitis B virus E antigen (HBeAg)-positive patients was observed, but no effect in virally suppressed patients was observed (20).

To further characterize the mechanism of CAM-A compounds and understand the discrepancy between the preclinical models and CHB patients, we performed a detailed profiling of a tool CAM-A, HAP_R10, an early structural analog of RG7907 (21), alongside the CAM-E GS-SBA-1P (11). Studies were performed utilizing HBV-infected PHH, the immune-competent adeno-associated virus (AAV)-HBV mouse model, and the immune-deficient liver chimeric HBV-infected urokinase-type plasminogen activator/severe combined immunodeficient mouse model (uPA-SCID) model. Both molecules stimulated potent reduction of HBV DNA, but only HAP_R10 induced nuclear HBc aggregation both *in vitro* and *in vivo*. Despite the formation of HBc aggregates, we did not observe any evidence of cell death in HBV-infected PHH. Nevertheless, consistent with previous reports (16) and further clarifying that adaptive immunity is not the primary driver of CAM-A-induced HBc-positive cell death, HBsAg reduction and loss of HBc-positive cells were observed in both HBV mouse models, albeit with different kinetics and magnitude of HBsAg decline. To better understand the differences in the effect of CAM-A on HBc-positive cell viability, we assessed the levels of HBc protein across these model systems and compared this to CHB patient liver biopsies. Our findings demonstrate that baseline HBc protein levels are positively correlated with CAM-A-induced killing of HBc-positive cells which has important implications for the translatability of these preclinical findings to the clinic.

## RESULTS

### HAP_R10 is a potent inhibitor of HBV DNA production in PHH

**Fig 1:**
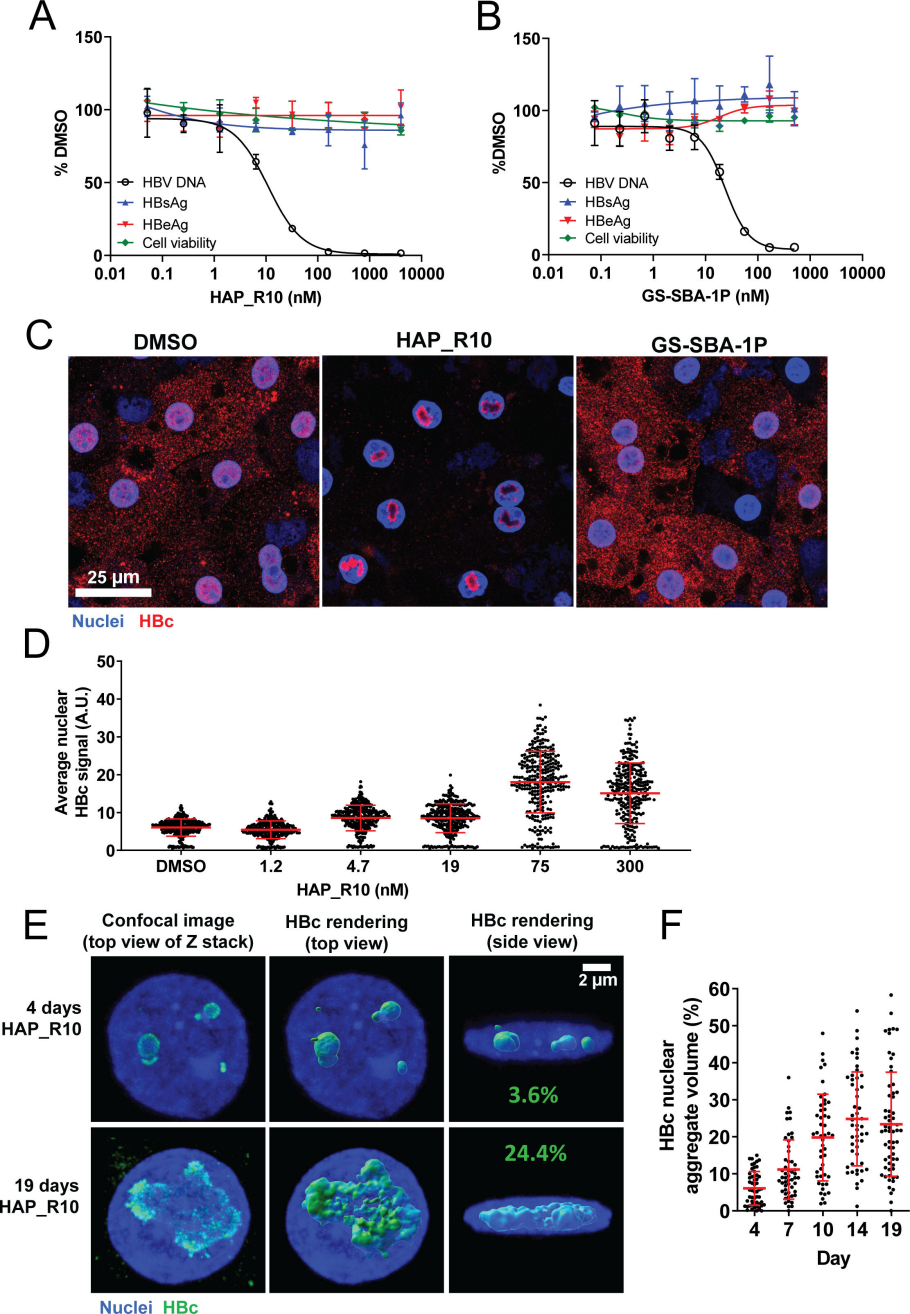
HAP_R10 reduces HBV DNA and promotes nuclear HBc aggregation in HBV-infected PHH. Representative dose-response curves of extracellular HBV DNA, HBsAg, HBeAg, and cell viability in HBV-infected PHH treated with either (**A**) HAP_R10 or (**B**) GS-SBA-1 for 6 days after the drugs were added at day 3 post infection. Data are shown as the percent relative to DMSO-only control with mean ± SD. (**C**) Representative confocal images of HBV-infected PHH stained for HBc (red) and nuclei (blue) following 17 days of either 20X EC_50_ HAP_R10 or GS-SBA-1P treatment. (**D**) Nuclear HBc signal in HBV-infected PHH treated with different concentrations of HAP_R10. Data are shown as HBc signal per individual nuclei with mean ± SD. (**E**) Representative confocal images and 3D rendering of HBV-infected PHH. HBc aggregates were used for volumetric measurements after treatment with 300 nM HAP_R10 for either 4 days (top row) or 19 days (bottom row). Percentages shown on images with lateral views of the nuclei reflect a portion of the nuclear volume occupied by HBc aggregates. (**F**) A portion of the nuclear volume occupied by HBc aggregates following the time course of treatment with 300 nM HAP_R10. Data are shown as HBc signal per individual nuclei with mean ± SD (*N* = 49–62 nuclei).

The antiviral activity of HAP_R10 and GS-SBA-1P was evaluated in PHH 3 days post infection with HBV genotype D (Fig. 1; Table 1). Consistent with previous reports (11, 21), both compounds demonstrated potent activity against extracellular HBV DNA production (HAP_R10 EC_50_ = 0.017 ± 0.0013 µM and GS-SBA-1P EC_50_ = 0.012 ± 0.0012 µM) with no appreciable effect on cell viability or on the levels of secreted viral proteins [HBsAg and hepatitis B e antigen (HBeAg)], following 6 days of treatment (Fig. 1A and B; Table 1). In addition, HAP_R10 also demonstrated the potential to block cccDNA establishment in PHH when treated at the time of HBV infection. While the HAP_R10 potency for cccDNA establishment is lower than that of GS-SBA-1P (11), HAP_R10 reduced intracellular HBV RNA (EC_50_ = 1.4 µM) and HBV antigens, HBeAg (EC_50_ = 1.5 µM) and HBsAg (EC_50_ = 2.1 µM) with no measurable effect on the cellular viability (CC_50_ > 4.5 µM; Table S1).

**TABLE 1:** Antiviral activity of HAP_R10 and GS-SBA-1P following 6 days of treatment in HBV-infected PHH^*a*^

	Viral DNA EC_50_ (µM)	HBsAg EC_50_ (µM)	HBeAg EC_50_ (µM)	Cytotoxicity CC_50_ (µM)
HAP_R10	0.017 ± 0.0013 (*n* = 16)	>2	>2	>2
GS-SBA-1P	0.012 ± 0.0012 (*n* = 6)	>2	>2	>2

^*a*^ Mean values ± SD are shown; *n* = number of independent experiments.

### HAP_R10 promotes nuclear HBc aggregation in HBV-infected PHH

Consistent with previous studies, treatment with HAP_R10, but not GS-SBA-1P, induced nuclear HBc aggregates in HBV-infected PHH at concentrations 20× EC_50_ (Fig. 1C). We selected this concentration for the compound treatment as it provides sufficient target coverage without off-target effects associated with an excessive compound dosage. HAP_R10 presence at the site of aggregation was confirmed using a fluorescently labeled version of the compound with retained ability to form HBc aggregates (Fig. S1A and B). Nuclear accumulation of HBc occurred in a dose-dependent manner (Fig. 1D) and expression of HBc alone in PHH transfected with HBc mRNA was sufficient to induce aggregate formation (Fig. S2). Compound washout following a 3-day treatment with 20× EC_50_ (300 nM) HAP_R10 led to the disappearance of the HBc aggregates and to a considerable decrease in the ratio of the HBc-specific signal in nucleus vs cytoplasm confirming that the HBc aggregation in HBV-infected PHH is reversible (Fig. S3). Notably, the spatial distribution of HBc within the nucleus changed from aggregates in central areas to peripheral localization (Fig. S3A). Volumetric measurements based on 3D reconstructions revealed that the nuclear volume occupied by HBc aggregates increased over time plateauing at approximately 20%–30% by day 10 after the onset of treatment (Fig. 1E and F). In contrast to recent reports with CAM-As in HBV-integrated cancer cell lines (14, 15), we did not observe any evidence of cell death in HBV-infected PHH with up to 38 days of HAP_R10 treatment (Table S2). In addition, we also did not observe any evidence of cell death following long-term treatment of HAP_R10 in HepAD38 cells or HBV-infected HepG2-NTCP cells despite confirming the formation of HBc aggregates (Table S2; Fig. S4).

### HAP_R10 treatment induces the elimination of HBc-positive hepatocytes in the AAV-HBV mouse model

CAM-A inhibitors have previously reported a strong and sustained decline of viral markers coinciding with the loss of HBc-positive cells in the immunocompetent AAV-HBV mouse model (14–16, 22–24). This widely used model is based on AAV-mediated delivery of the HBV genome into immunocompetent C57BL/6 mice (25). It achieves a persistent HBV infection with high levels of viral antigens and DNA without significant inflammation or elevation of serum alanine aminotransferase (ALT) in the liver (26, 27).

**Fig 2:**
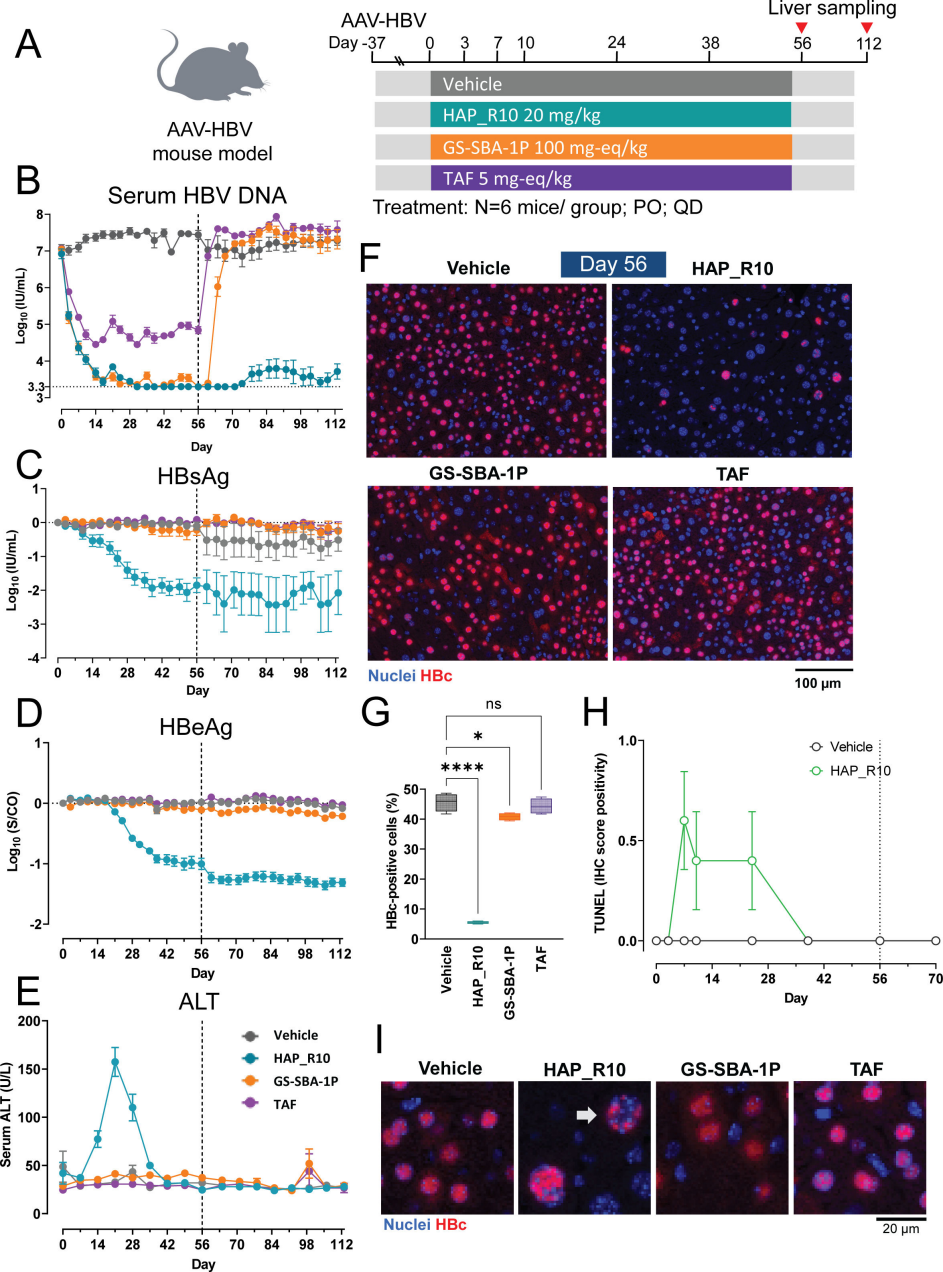
HAP_R10 treatment induces elimination of HBc-positive hepatocytes in the AAV-HBV mouse model. (**A**) Schematic of the HAP_R10, GS-SBA-1P, and TAF efficacy study in AAV-HBV mice. Mice injected with AAV-HBV for 37 days were dosed with 20 mg/kg HAP_R10, 100 mg-eq/kg GS-SBA-1P, and 5 mg-eq/kg TAF once daily for 56 days. Mice were followed for an additional 56 days. Longitudinal analysis of (**B**) serum HBV DNA, (**C**) HBsAg, (**D**) HBeAg, and (**E**) ALT levels during treatment and after treatment follow-up. All data are presented as mean ± SD. (**F**) Representative immunofluorescence images of the liver tissue slices stained for HBc and nuclei at day 56 of treatment. (**G**) Box (extends from the 25th to 75th percentiles) and whiskers (Min–Max) plot showing the percentage of HBc-positive cells in samples from day 56 of the study. Statistical analysis was performed using one-way ANOVA *t* test. ****, *P* < 0.0001; *, *P* < 0.05; ns, not significant *P* > 0.05. (**H**) Time course of the terminal deoxynucleotidyl transferase dUTP nick-end labeling (TUNEL) signal in liver samples from animals dosed with vehicle or HAP_R10. Immunohistochemistry (IHC) staining positivity scores: 0, negative; 1, positive staining cells <25%. (**I**) Enlarged areas of images similar to those shown in Fig. 2F and selected to demonstrate HAP_R10-induced formation of HBc aggregates in tissue samples.

To verify that CAM-A phenotype also occurs with HAP_R10 in the AAV-HBV mouse model, we profiled the *in vivo* efficacy of HAP_R10 in parallel with the CAM-E GS-SBA-1P. Mice were injected with 1 × 10^11^ viral genomes of either AAV-HBV or an empty AAV vector. Treatment with 20 mg/kg HAP_R10, 100 mg-eq/kg GS-SBA-1P, or 5 mg-eq/kg of the NA tenofovir alafenamide (TAF) was started 37 days post AAV injection when the infection reached a steady state. Mice were dosed orally once a day for 56 days and followed for an additional 56 days post-treatment (Fig. 2A). The concentrations of HAP_R10 measured in the plasma and liver demonstrated a high steady-state trough concentration of HAP_R10 in the liver, which was approximately eightfold above the protein-adjusted (pa) EC_50_ of HAP_R10 (paEC_50_ = 68 nM, Fig. S5; Table S3). Plasma and liver concentrations of GS-SBA-1P and TAF were similar to those previously reported, achieving approximately 76-fold over the paEC_50_ and human equivalent exposure, respectively (11).

Serum HBV DNA, viral antigens (HBeAg and HBsAg), and ALT levels were measured weekly throughout the study, and livers were collected from animals at various timepoints until terminal sacrifice at day 112 post-treatment. By the end of the treatment (day 56), HAP_R10, GS-SBA-1P, and TAF significantly (*P* < 0.0001) reduced serum HBV DNA levels (3.7 log_10_, 3.7 log_10_, and 2.5 log_10_, respectively; Fig. 2B). Consistent with published results for RG7907, HAP_R10 induced >2 log_10_ HBsAg reduction (*P* < 0.0001) and ~1 log_10_ HBeAg reduction (*P* < 0.0001) relative to vehicle by the end of the treatment (Fig. 2C and D). No rebound of any of the measured viral parameters was observed after the end-of-treatment, consistent with the lack of viral spread in this model (Fig. 2B through D). The onset of HBsAg and HBeAg reduction coincided with a transient ALT flare, peaking at day 21 of HAP_R10 treatment (Fig. 2E). The changes in HBsAg and ALT were delayed compared to rapid reduction in serum HBV DNA, indicating a difference in the underlying processes. No ALT increase was observed with HAP_R10 in mice that received only the AAV vector demonstrating that the response was HBV dependent (data not shown). There were no changes in HBsAg, HBeAg, or ALT in the presence of either GS-SBA-1P or TAF, and HBV DNA rebounded to baseline levels following the withdrawal of these compounds.

To determine whether the sustained reduction of viral parameters observed following cessation of HAP_R10 treatment was due to the loss of HBc-positive cells, immunofluorescence staining of mouse livers for HBc was performed. There was no change in the percentage of HBc-positive cells in the liver of animals treated with TAF and minimal reduction with GS-SBA-1P at the end-of-treatment (Fig. 2F and G). In contrast, HAP_R10-treated animals had a 90% reduction (*P* < 0.0001) of HBV-infected hepatocytes as measured by the remaining HBc-positive cells (Fig. 2F and G). Terminal deoxynucleotidyl transferase dUTP nick-end labeling (TUNEL) staining was performed on HAP_R10-treated livers over the course of the study. An increase in TUNEL was observed from day 7 to day 24 (Fig. 2H), coinciding with the transient ALT flare (Fig. 2E). These observations suggest that the depletion of the HBc-positive hepatocytes was due to cell death. Nuclear HBc aggregates were only observed in the HBc-positive hepatocytes from animals treated with HAP_R10 (Fig. 2I), consistent with the *in vitro* PHH data (Fig. 1C).

**Fig 3:**
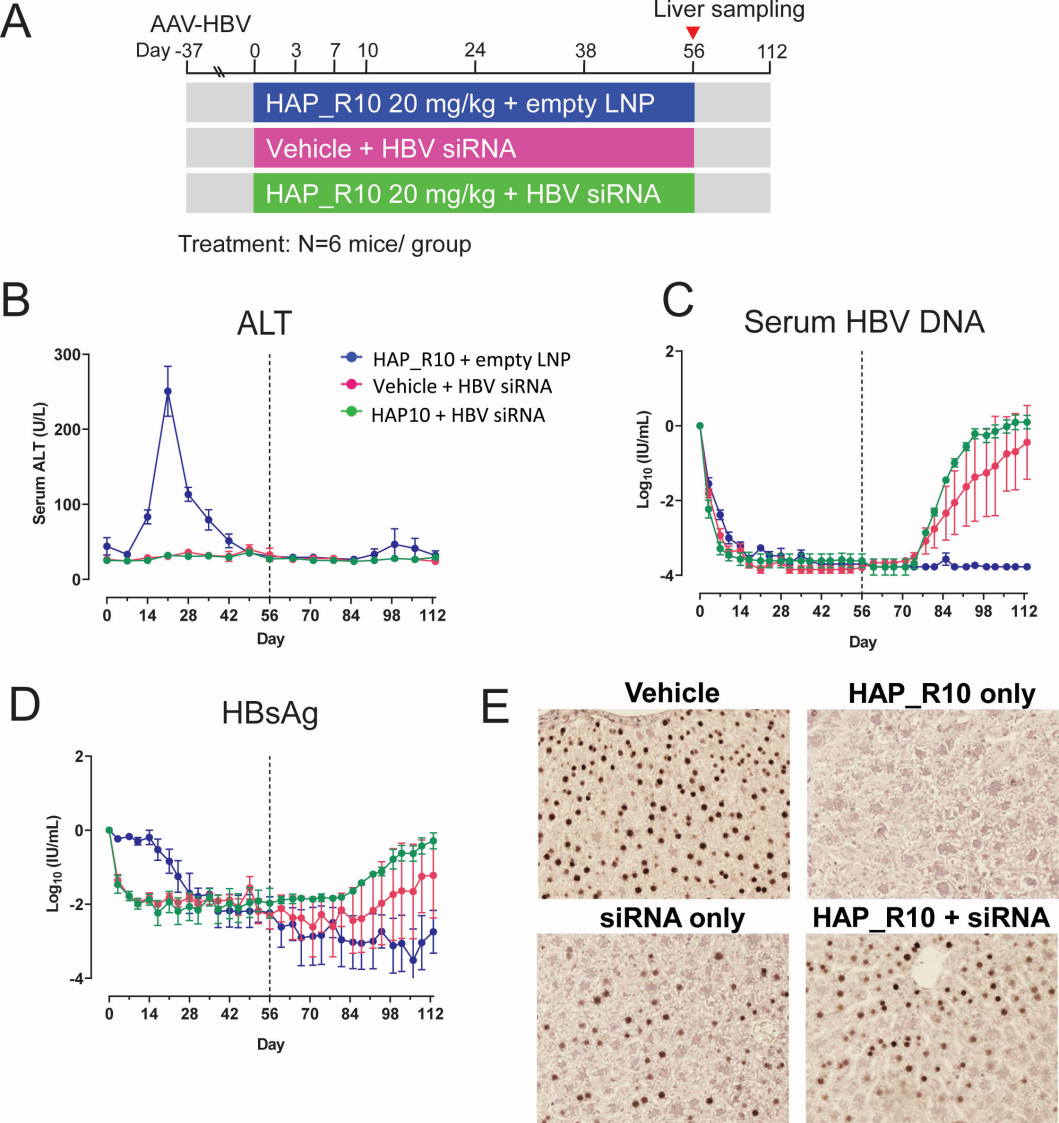
HAP_R10 HBc-positive cell killing phenotype is HBV-dependent in AAV-HBV mice. AAV-HBV mice were treated either with an HBx-targeting HBV siRNA or vehicle control followed by the start of treatment with 20 mg/kg HAP_R10 the same day. (**A**) Schematic of the HAP_R10 efficacy study in the presence of HBV-targeting siRNA. Longitudinal analysis of (**B**) serum ALT, (**C**) serum HBV DNA, and (**D**) HBsAg. All data are presented as mean ± SD. (**E**). Representative immunohistochemistry images of liver tissue samples were collected on the last day of treatment and stained for HBc.

To confirm that the *in vivo* HAP_R10 phenotype is HBV-dependent, AAV-HBV mice were either treated with an HBx-targeting HBV siRNA dosed at 3 mg/kg IV every 2 weeks (28) or injected with empty liponanoparticles (LNPs) coinciding with the start of HAP_R10 treatment (Fig. 3). By day 7, HBV siRNA reduced HBV DNA (3.3 log_10_ IU/mL) and HBsAg (1.8 log_10_ IU/mL). No HAP_R10-induced ALT elevation or sustained depletion of HBsAg or DNA was observed in the presence of the HBV siRNA, confirming that HAP_R10-induced elimination of hepatocytes requires expression of HBV proteins (presumably HBc) and not just the presence of the HBV genome (Fig. 3B through E).

### HAP_R10 decreases levels of HBsAg in the absence of an adaptive immune response in an immunocompromised HBV chimeric mouse model

**Fig 4:**
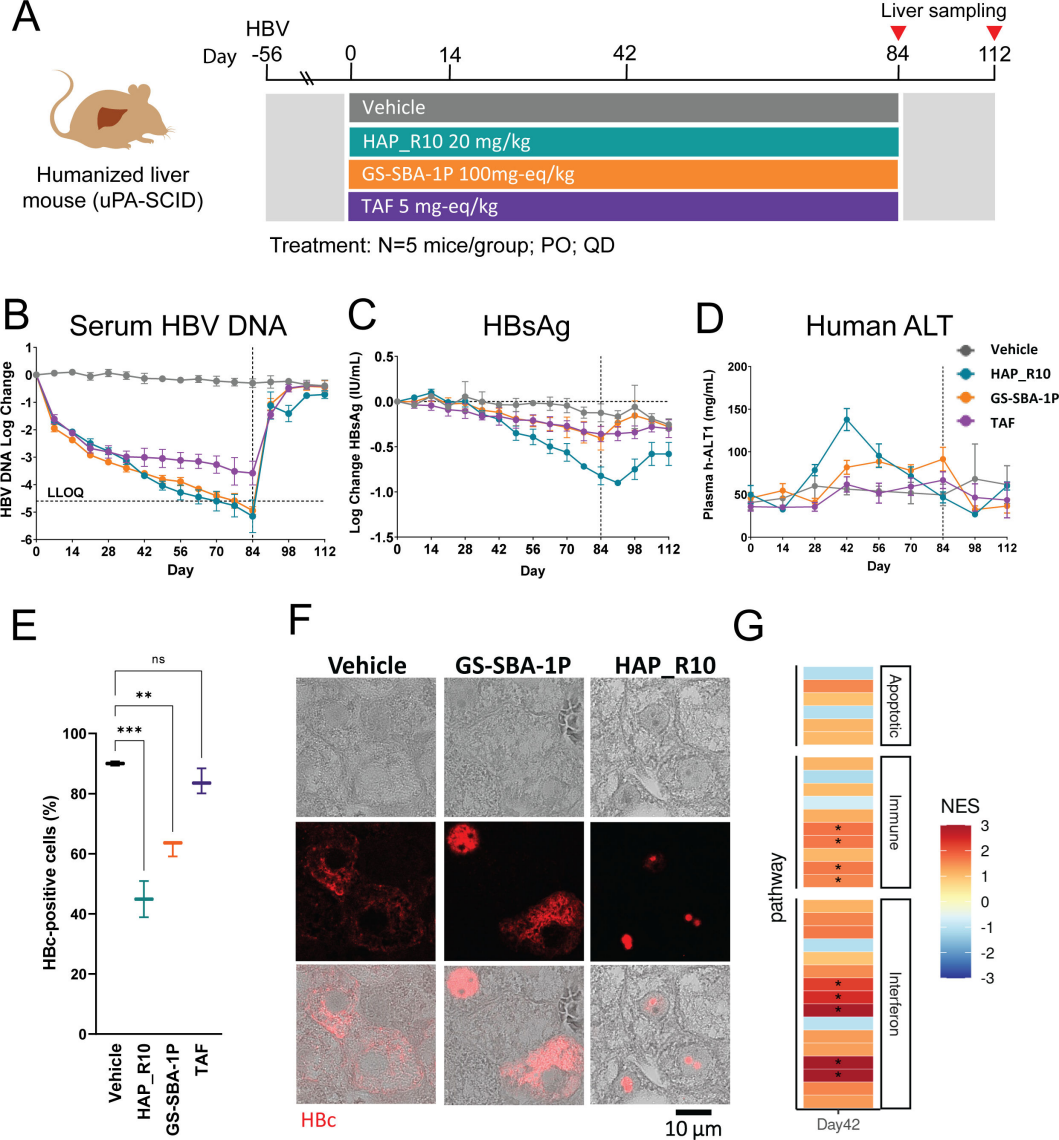
HAP_R10 decreases the levels of HBsAg in the absence of an adaptive immune response in an immunocompromised HBV chimeric mouse model. (**A**) Schematic of the HAP_R10, GS-SBA-1P, and TAF efficacy study in uPA-SCID mice. After 8 weeks of infection, mice were dosed with 20 mg/kg HAP_R10, 100 mg-eq/kg GS-SBA-1P, or 5 mg-eq/kg TAF (*n* = 5 per group) for 84 days. Mice were followed for an additional 28 days. Longitudinal analysis of (**B**) serum HBV DNA, (**C**) HBsAg, and (**D**) human ALT levels during treatment and after treatment follow-up. All data are presented as mean ± SD. LLOQ stands for the lower limit of quantification. (**E**) Box (extends from the 25th to 75th percentiles) and whiskers (Min–Max) plot showing the percentage of HBc-positive cells in samples from day 84 of the study. Statistical analysis was performed using one-way ANOVA *t* test. ***, *P* < 0.001; **, *P* < 0.01; ns, not significant *P* > 0.05. (**F**) Representative transmission and immunofluorescence images of the liver tissue slices stained for HBc (red). (**G**) Gene Set Enrichment Analysis (GSEA) results showing gene sets associated with apoptotic, immune, and interferon-related pathways from KEGG, REACTOME, and HALLMARK gene sets. GSEA normalized enrichment scores (NES) are denoted by color bar and reflected changes relative to HBV-infected day 42 vehicle-treated mice. Boxes containing asterisks are pathways with a false discovery rate (FDR) < 0.05.

A major limitation of AAV-HBV mouse model is that murine hepatocytes are not infected by HBV, and therefore, this model does not allow for viral spread (27). To address this limitation and evaluate the potential involvement of the adaptive immune response, we profiled HAP_R10 in the humanized liver chimeric uPA-SCID. This model closely mimics human HBV infection and can support a long-term persistent infection with the full HBV replication cycle (including viral spread) but lacks a functional adaptive immune system. Mice (*n* = 20) were infected with HBV genotype C virus. After viral kinetics reached a steady state (day 56), animals were orally dosed once daily with 20 mg/kg HAP_R10, 100 mg-eq/kg GS-SBA-1P, or 5 mg-eq/kg TAF for 84 days. Mice were then monitored for an additional 28 days (Fig. 4A). Serum samples were collected weekly to monitor HBV viral antigens, HBV DNA, and ALT. Similar liver and blood exposures to the AAV-HBV mouse model were achieved for all three compounds.

Consistent with the AAV-HBV mouse model, HAP_R10, GS-SBA-1P, and TAF all strongly reduced serum HBV DNA levels, with a mean decrease of 5.2 log_10_ (*P* < 0.0001), 5.0 log_10_ (*P* < 0.0001), and 3.6 log_10_ (*P* < 0.0001), respectively, compared to the vehicle (Fig. 4B). A significant decline in HBsAg was observed with HAP_R10 treatment relative to baseline (0.8 log_10_, *P* < 0.05, day 84 vs day 0), which coincided with the peak of the ALT flare (*P* < 0.0001, day 42 vs day 0; Fig. 4C and D). However, the onset of ALT was delayed, and the overall magnitude of HBsAg decline was reduced compared to the AAV-HBV mouse model. Modest, although statistically significant changes in HBsAg were also observed for GS-SBA-1P (0.4 log_10_, *P* < 0.05) and TAF (0.35 log_10_, *P* < 0.01) at day 84 vs day 0,_,_ respectively, but these were relatively minor compared to HAP_R10. Following cessation of treatment with GS-SBA-1P and TAF, all HBV parameters returned to similar levels as vehicle-treated mice (Fig. 4B and C). In HAP_R10 treated animals, a partial rebound of both serum HBV DNA and HBsAg levels following treatment cessation was observed, remaining at – 0.7 log_10_ (*P* = 0.001) and – 0.6 log_10_ (*P* > 0.05) from baseline, respectively. Consistent with this observation, immunofluorescence staining of liver tissues from HAP_R10 treated mice demonstrated a 50% reduction of HBc-positive cells compared to vehicle or TAF-treated animals. Surprisingly, we observed a 30% reduction of HBc-positive cells in mice treated with GS-SBA-1P despite the viral parameters rebounding to similar levels as vehicle following treatment cessation (Fig. 4E). GS-SBA-1P has previously been shown to block cccDNA establishment at concentrations six-fold over HBV DNA the plasma-adjusted EC_50_ (11). Considering plasma exposures reached greater than 76-fold the mouse plasma-adjusted EC_50_ of HBV DNA production, we hypothesize that GS-SBA-1P prevented new infection and cccDNA formation following hepatocyte turnover. Of the remaining HBc-positive cells, confocal imaging confirmed the formation of HBc aggregates in the presence of HAP_R10 but not GS-SBA-1P (Fig. 4F). Together these results suggest that the HBsAg decline associated with ALT flare observed in the HAP_R10-treated immunodeficient uPA-SCID mice does not require the adaptive immune response and may be a result of cell death induced by HAP_R10-mediated HBc aggregates formation.

### HAP_R10 induces innate inflammatory responses and activation of genes associated with apoptotic pathways in both HBV mouse models

To further elucidate the HAP_R10 mechanism of cell death observed *in vivo*, we performed RNA-Seq analysis of livers from both the AAV-HBV and HBV-infected uPA-SCID mice treated with either HAP_R10 or vehicle. In AAV-HBV mice, liver samples were collected at multiple time-points during and after treatment (days 3 through 70; Fig. 2A). In the AAV-HBV mouse model, the number of differentially expressed genes increased by day 7 [*n* = 34, false discovery rate (FDR) < 0.05 and log fold change (|LFC)| > 1)], peaking at the height of the ALT flare on day 24 (*n* = 529, FDR < 0.05, |LFC| > 1). We observed upregulation of individual genes within cell proliferation (G2M checkpoint and EF2 targets), interferon, mitotic, and apoptotic pathways in AAV-HBV mouse model at days 7 through 24 and returning to baseline as ALT resolved (14) (Fig. S6A). Also, no adaptive immunity gene pathways were induced with HAP_R10 treatment which is consistent with demonstrating efficacy in the uPA-SCID mouse model.

Gene expression profiling was conducted in the uPA-SCID mouse model at the peak of ALT flare (day 42) and revealed significant activation (FDR < 0.05) of both innate immune and interferon-associated pathways, consistent with the interferon response induction in the AAV-HBV mouse model. While not statistically significant (FDR = 0.16), the apoptotic pathway was moderately upregulated [nominal *P* < 0.05; Gene Set Enrichment Analysis (GSEA) normalized enrichment scores = 1.5; Fig. 4G; Fig. S6B; Table S4]. Intrahepatic serum amyloid A genes (FDR < 0.01) were significantly upregulated in both AAV-HBV and uPA-SCID models (Fig. S6C), consistent with the activation of a common inflammatory response. These results suggest that treatment with HAP_R10 was associated with stimulation of an innate inflammatory response and upregulation of genes associated with apoptosis at the time of ALT elevation. The upregulation of apoptotic genes is consistent with results of the TUNEL assay.

### Autophagy-related protein p62 regulates HAP_R10-induced HBc aggregate size

Nuclear HBc aggregation is a key phenotypic differentiator between CAM-As and CAM-Es. Therefore, we sought to investigate potential mechanisms that can modulate the formation of CAM-A-induced HBc aggregates. It has been previously shown that U box-containing protein 1 (STUB1) can promote p62-mediated macroautophagy and lysosomal degradation of CAM-A-induced HBc aggregates (29). The ubiquitin-binding protein p62 (also referred to as sequestosome-1, SQSTM1) plays a key role in the degradation of nuclear protein aggregates (30, 31). Interaction with the tripartite motif-containing protein 16 with E3 ubiquitin ligase activity (TRIM16) is required for p62 for assembly of these protein aggregates for degradation.

**Fig 5:**
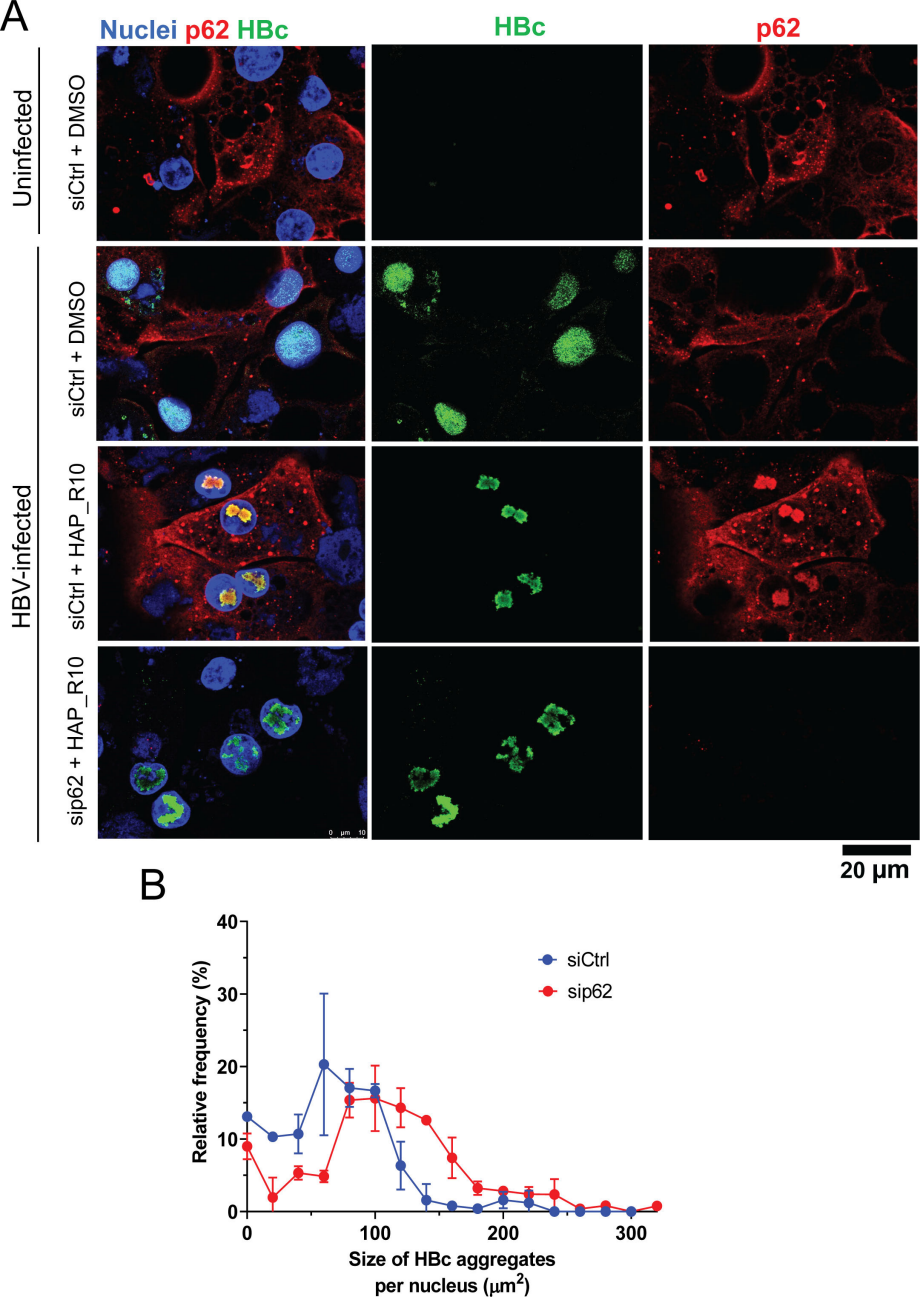
Autophagy-related protein p62 regulates HAP_R10-induced HBc aggregate size. (**A**) Representative confocal images of uninfected and HBV-infected PHH treated with 20× EC_50_ (300 nM) HAP_R10 or DMSO for 14 days in the presence of siRNA-mediated knockdown of p62 or siRNA control (siCtrl). PHH was stained for HBc (green), p62 (red), and nuclei (blue). (**B**) Frequency distribution of the combined size of the HBc aggregates per nucleus. Data are shown as mean ± SD (*n* = 2 independent experiments).

To investigate if p62 and TRIM16 affect the degradation of HAP_R10-induced HBc aggregates, immunofluorescence and colocalization analysis were performed in uninfected and HBV-infected PHH. In control PHH cells treated with dimethyl sulfoxide (DMSO), p62, and TRIM16 were primarily localized in the cytoplasm regardless of the infection status of the cells. Upon HAP_R10 treatment, both p62 and TRIM16 translocated to the nucleus colocalizing with the HBc aggregates (Fig. 5A; Fig. S7). Attempts to knockdown TRIM16 using siRNA were unsuccessful (data not shown), but knockdown of p62 was confirmed by immunofluorescence (Fig. 5A). In HBV-infected PHH treated with HAP_R10, knockdown of p62 led to a moderate increase in the size of the HBc aggregates per nucleus (Fig. 5B; fold change = 1.51 ± 0.16 relative to siCtrl transfection), suggesting that p62 may play a role in the degradation of HBc aggregates. Knockdown of p62 in DMSO-treated HBV-infected PHH resulted in HBc accumulation predominantly at the periphery of the nucleus (Fig. S8). This finding is consistent with previous studies demonstrating aggregation of HBc to the peri-nuclear region in the presence of an inhibitor of macroautophagy, 3-MA. The data may indicate a potential role of p62 in trafficking HBc (possibly in excess of HBc protein or aggregates) in the absence of CAMs (29).

Furthermore, RNA-Seq analysis of p62/SQSTM1 expression in the AAV-HBV mouse efficacy study demonstrated a significant, but moderate, induction in p62 (sqstm1) expression starting at day 7 (log2 Fold Change 0.23 padj = 0.036) post-HAP_R10 treatment until the peak of ALT elevation at day 24 (log2 Fold Change 0.44 padj = 7.61E-04; Table S5). No significant changes in p62 expression level in the uPA-SCID mouse efficacy study were detected at the peak of ALT elevation on day 42 (log2 Fold Change −0.104 padj = 0.63) which could be attributed to the reduced CAM-A induced HBc-positive cell death in this model vs the AAV-HBV.

### CAM-A-mediated reduction of HBc-positive cells correlates with higher baseline levels of HBc

**Fig 6:**
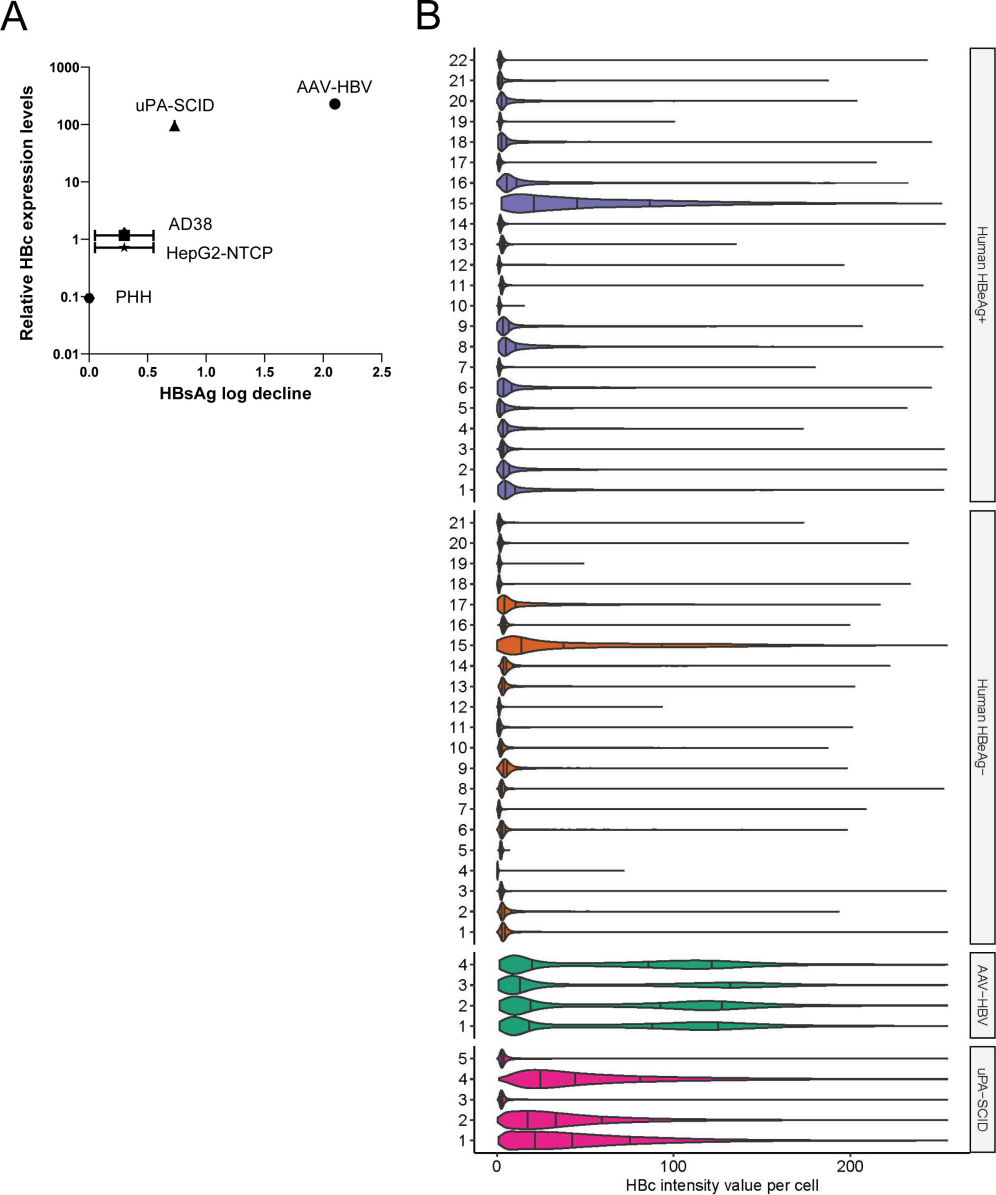
CAM-A-mediated reduction of HBV-positive cells correlates with higher baseline levels of HBc. (**A**) Correlation plot of HBc expression levels determined by western blot analysis compared to HBsAg decline across the different animal and cell culture models. Data are shown as mean ± SD. (**B**) Violin plots showing the distribution of average HBc intensity per cell in CHB HBeAg(+) and HBeAg(−) patients and AAV-HBV and HBV-infected uPA-SCID mice.

We hypothesized that the differences in the magnitude of HAP_R10-mediated reduction of HBV-positive cells in the two *in vivo* models, the failure of HAP_R10 to eliminate HBV-positive hepatocytes in AAV-HBV mice treated with HBV siRNA while still retaining similar activity against extracellular DNA (Fig. 3), and the failure to kill HBV-infected PHH and HBc-positive cell lines *in vitro* following long-term treatment (Table S2), may be due to differences in HBc expression levels. To test this hypothesis, we compared the total levels of HBc across the different animal and cell culture models by western blot analysis (Fig. 6A). The analysis revealed a positive association between the level of HBc expression and the magnitude of the HAP-R10-induced reduction of extracellular HBsAg (Pearson’s *r* = 0.97, *P* < 0.001). The AAV-HBV mouse model had the highest levels of HBc protein, while the *in vitro* cell cultures had the lowest levels of HBc protein. As the western blot does not account for differences in infectivity, we assessed the variability in the HBc levels on an individual cell basis using multiplex immunofluorescence staining on liver sections of both the HBV-infected uPA-SCID and AAV-HBV models. The highest HBc levels were observed in the AAV-HBV (average HBc intensity per cell 69.4 ± 43.5) followed by the HBV-infected uPA-SCID mouse model (36.7 ± 43.5) which correlates with the degree of HBc-positive cell death. We performed the same analysis on baseline liver biopsies from a range of HBeAg-positive (*n* = 22) and HBeAg-negative (*n* = 21) CHB patients from clinical studies GS-US-174–0102 (ClinicalTrials.gov: NCT00117676) and GS-US-174–0103 (ClinicalTrials.gov: NCT00116805; Table S6; Fig. 6B). Overall, there was a wide range of HBc levels in the mouse models and CHB patient biopsies, although mean HBc levels were lower in CHB patient samples (average HBc intensity per cell 10.1 ± 24.6 and 8.83 ± 18.4 in HBe− and HBe+ patients, respectively) compared to the preclinical models. In addition, no specific measured virological markers [HBsAg, HBV DNA, HBeAg status, and HBV core-related antigen (HBcrAg)] were found to correlate in CHB patients with higher levels of intracellular HBc. Given the HBc levels in CHB patient biopsies are lower than even the uPA-SCID mouse model (in which there was only modest efficacy), it is likely that little-to-no CAM-A-induced cell death would be observed in the majority of CHB patients.

## DISCUSSION

The majority of CHB patients, even following long-term NA therapy, are unable to achieve functional cure as defined by sustained HBsAg loss. Therefore, new therapies are actively being developed. CAM-As have shown a significant reduction of HBV-infected hepatocytes in preclinical mouse models; however, they have had limited success to date in CHB patients (14, 16, 18–20, 22–24). Here, we provide a detailed characterization of the antiviral activity of the CAM-A HAP_R10, its mechanism of action through nuclear HBc aggregate formation, and the translatability of the HBV preclinical animal models to CHB patients.

One major difference between the CAM-As and CAM-Es is the formation of nuclear core aggregates (14–16, 32, 33). In this study, we demonstrated that treatment with HAP_R10 induced large HBc-aggregates in nuclei of HBV-infected PHH, HepAD38 cells with a complete HBV genome, and in PHH transfected with HBc mRNA. The size of the aggregates observed in HBV-infected PHH gradually increased, reaching 50% of the nuclear volume following 19 days of the treatment. It has been hypothesized that these HBc nuclear aggregates trigger the elimination of HBV-infected hepatocytes (33). Evidence to support this hypothesis was obtained from studies with RG7907 in cancer cell lines overexpressing HBc (14, 16). However, in our study, HAP_R10-induced formation of HBc aggregates had no negative impact on cell viability with the long-term treatment of HBV-infected PHH or the cancer cell lines, HepAD38, and HBV-infected HepG2-NTCP. We hypothesized that the HBc expression levels were not sufficient to stimulate cell death in cell culture within the duration tested due to lower HBc protein levels.

Unlike *in vitro*, the formation of HAP_R10-induced HBc aggregates observed in both the uPA-SCID and the AAV-HBV mouse livers was associated with the elimination of HBc-positive cells. Despite achieving similar compound exposure, the elimination of HBc-positive hepatocytes was more pronounced in the AAV-HBV mouse model (approximately eightfold decline) than in the HBV-infected immune-deficient uPA-SCID mice (approximately twofold decline) consistent with the recent findings by Berke et al. Similar to the findings in the AAV-HBV mouse model by others (14, 16), we also observed a significant increase in the expression of liver genes associated with cell proliferation, interferon response, and apoptosis. In the uPA-SCID model, a similar trend in gene signature is observed, but response is more muted and does not achieve statistical significance likely due to the reduced CAM-A-induced HBc-positive cell death in this model vs the AAV-HBV. These data further confirm the lack of association between an adaptive immune response and HBc-positive cell death. Presumably, aggregate formation triggers this response, but it is unclear how as we observe no evidence of the unfolded protein response or other stress signatures.

There has not been a substantial HBsAg reduction reported to date in CHB patients treated with either RG7907 or GLS4, despite both compounds strongly reduce serum HBV DNA and HBV RNA levels (18–20). This is likely due, at least in part, to the production of HBsAg from HBV-integrated cells, which do not express HBc and therefore are not expected to be susceptible to CAM-A treatment (34, 35). Our study provides an additional explanation for the discrepancy in preclinical and clinical efficacy of CAM-A. We identified a positive correlation between intracellular HBc levels and CAM-A-induced HBc-positive hepatocyte killing and provided the first piece of evidence that directly measures intracellular concentrations of HBc. Lower levels of intracellular HBc potentially restrict the formation of aggregates leading to limited impact on the cell viability. Compared to the AAV-HBV mouse model, killing of HBc-positive cells is significantly diminished in the uPA-SCID mouse model which has ~50% less intracellular levels of HBc. As intracellular HBc levels in CHB patient biopsies are much lower than in the HBV mouse models, we suspect only a minimal amount of CAM-A killing would be achievable.

In summary, our data demonstrate that while CAM-As have the potential to induce cell death of HBc-positive hepatocytes, the level of cell killing is dependent on baseline levels of intrahepatic HBc. Furthermore, we demonstrated that one of the factors restricting the size of the HBc aggregates is p62-mediated removal of misfolded/aggregated HBc in the nucleus which could negatively impact CAM-A clearing of HBc-positive cells. Profiling of HBc levels per hepatocyte across CHB patient biopsies revealed much lower levels compared to the HBV animal models likely limiting HBc-positive cell killing in the clinic. Berke et al. had previously hypothesized that patients with higher levels of HBcrAg (>8 log_10_ U/mL) would have a greater likelihood of achieving this mechanism. However, in our studies, we found no correlation between HBcrAg and intracellular HBc in our CHB patient biopsies. Furthermore, we were not able to identify any other discerning serum virological features from CHB patients that had higher HBc intracellular levels to predict efficacy. Our findings raise important concerns regarding the translatability of CAM-A preclinical findings for achieving CHB cure in patients.

## MATERIALS AND METHODS

### Compounds

TAF, HAP_R10, GS-SBA-1P, and Alexa-tagged HAP_R10 were synthesized by Gilead Sciences Inc.

### Cell lines

HepAD38 cells were grown in DMEM-F12 medium (Thermo Fisher Scientific, 10565018) supplemented with 10% fetal bovine serum (FBS; Thermo Fisher Scientific), 1% penicillin-streptomycin-glutamine (Thermo Fisher Scientific, 10378016), 1% 4-(2-hydroxyethyl)−1-piperazineethanesulfonic acid (HEPES; Thermo Fisher Scientific, 15630080), and 1% non-essential amino acids (Thermo Fisher Scientific, 11140050).

### PHH culture conditions and HBV infection

Cryopreserved PHHs isolated from multiple donors were purchased from Thermo Fisher Scientific (Waltham, MA), Bioreclamation IVT (Hicksville, NY), or Lonza (USA). PHH culturing conditions as previously described (11). Approximately 24 h after plating, PHH was infected with HepAD38-derived HBV virions (36) (genotype D virus) at 500 viral genome equivalents per cell in a maintenance medium supplemented with 4% PEG 8000 (Promega, Madison, WI; V3011). The infection was allowed to proceed for 20–24 h before removing remaining extracellular virions by washing with a maintenance medium three times.

### Measurement of HBeAg and HBsAg concentrations by immunoassay

Extracellular HBsAg and HBeAg were detected in culture media by an electrochemiluminescence assay (Meso Scale Discovery [MSD]) as previously described (37).

### Quantification of extracellular HBV DNA

Extracellular HBV DNA from PHH supernatants was purified using the Qiagen DNeasy 96 kit (69582) following the protocol recommended by the manufacturer. Quantification of HBV DNA by qPCR amplification of the HBV X region of the genome was performed as previously reported (11).

### Immunostaining and confocal imaging

PHH was cultured on the glass coverslips (Corning BioCoat Poly-D-Lysine/Laminin 12 mm, Corning, NY) in 12-well Corning Cell bind plates. The cells were fixed with Perfusion Fixative Reagent (ThermoFisher Scientific) for 10 min and washed three times in Dulbecco’s Phosphate-Buffered Salt Solution (DPBS; Corning Cellgro 21–031-CV). Immunostaining was performed at 20°C–21°C. Permeabilization in 0.3% Triton X-100 (MilliporeSigma) for 15 min was followed by blocking in DPBS containing 3% bovine serum albumin (BSA) (MilliporeSigma A7030) and 10% HyClone FBS(MilliporeSigma) for 60 min. The final concentrations of primary antibodies were 0.5 µg/mL rabbit monoclonal HBV core antigen antibody (clone 366–2) made by Gilead Inc., mouse anti-HBV Core antibody (Abcam, ab8637, 1:100 dilution), 5.13 µg/mL mouse anti-HBsAg (XTL17 and IgG2b) made by Gilead Inc (38, 39), mouse monoclonal anti-SQSTM1/p62 antibody (Abcam, ab56416, 1:100 dilution), and rabbit anti-TRIM16 antibody (MilliporeSigma, HPA066431, 1:67 dilution). Secondary antibodies conjugated with either Alexa Fluor 488 (ThermoFisher Scientific, A21202, A11034) or Alexa Fluor 647 (ThermoFisher Scientific, A32733, A31571) were used at 2 µg/mL. All antibodies were diluted in DPBS containing 1.5% BSA. Primary and secondary antibodies were applied for 90 min and 60 min, respectively. The coverslips with stained cells were mounted on the glass microscopic slides (VWR International, Radnor, PA) with a drop of ProLong Diamond antifade reagent containing DAPI (ThermoFisher Scientific, P36962).

The samples were imaged with a confocal laser scanning microscope Leica SP8 (Leica Microsystems Inc., Wetzlar, Germany). All images within each sample set were captured using identical instrument settings. The acquisition was performed in separate sequences for each spectral channel to minimize bleed-through artifacts. DAPI was excited at 405 nm with UV laser and detected at 415–480 nm during the first sequence. Alexa Fluor 488 was excited at 488 nm and detected at 498–600 nm during the second sequence. Alexa Fluor 647 was excited at 647 nm and detected at 667–800 nm. Image analysis was performed in Imaris software, version 9.5.1 (Bitplane, Belfast, UK).

### AAV-HBV mouse model

Recombinant AAV (serotype 8) carrying the 1.3 copy HBV genome (genotype D; serotype ayw) and empty AAV were provided by Beijing FivePlus Molecular Medicine Institute (Beijing, China). C57BL/6 mice were provided by Shanghai Lingchang Bio Tech Co. Ltd (Shanghai, China). Studies were conducted by Covance Pharmaceutical R&D (Shanghai, China). All procedures implemented during the studies followed local animal welfare legislation, Covance global policies and procedures, and the Guide for the Care and Use of Laboratory Animals.

C57BL/6 mice (male; 4–5 weeks of age) were injected with 200 µL PBS containing 1 × 10^11^ vge recombinant virus per mouse through the tail vein. Twenty-eight days after AAV-HBV injection, the mice were bled retro-orbitally to monitor HBsAg and HBV genomic DNA in serum. Based on the HBsAg, HBeAg, and HBV DNA levels and body weight, AAV-HBV-infected mice were selected and randomized into groups (six animals per group) for compound and/or siRNA treatment. Vehicle, HAP_R10 20 mg/kg, GS-SBA-1P 100 mg/kg, and TAF 5 mg/kg were dosed by oral gavage once daily at 5 mL/kg for 56 days. LNP empty and LNP siRNA 3 mg/kg were injected intravenously at 4 mL/kg on dosing days 0, 14, 28, 42, and 56. HBV siRNA 77 (5' A.C.C.U.C.U.G.C.C.U.A.A.U.C.A.U.C.U.C.U.U 3') was produced by Dharmacon and packaged in LNPs by Precision Nanosystems at 0.75 mg/mL (28). On dosing days 0, 14, 28, 42, and 56, vehicle and HAP_R10 were administrated at 30 minutes following injections of LNP empty and LNP siRNA. Serum HBsAg, HBeAg, and HBV DNA were measured twice weekly. ALT was measured once weekly throughout the study. Serum HBsAg and HBeAg were determined by ARCHITECT i2000 (Abbott Laboratories, Lake Bluff, IL, USA). Serum HBV-DNA was analyzed by ABI7500 (Applied Biosystems, Foster City, CA, USA) and a detection kit (Sansure Biotech Inc., Changsha, Hunan, China). Serum ALT was determined using Roche Cobas 6000 Chemistry Analyzer (Roche Diagnostics, Mannheim, Germany) and supporting reagents.

### uPA-SCID mouse model

Male uPA-SCID mice between 12 and 18 weeks of age with humanized liver [cDNA-uPA^wild/+^/SCID (cDNA-uPA^wild/+^: B6;129SvEv-Plau, SCID:C.B-17/Icr-scid /scid Jcl] were produced as previously described by PhoenixBio, Co. Ltd., Japan (40). Briefly, frozen human hepatocytes (donor BD195, Corning Incorporated, Tewksbury, MA, USA) were thawed and transplanted into 2- to 4-week-old uPA/SCID mice by splenic injection. Mice were selected for studies if their liver reconstitution levels had an estimated replacement index greater than 70% based on the blood concentration of human albumin (>8.5 mg/mL) 1 week prior to study initiation. General health observations including weight were monitored weekly. All animal protocols were performed in accordance with the Guide for the Care and Use of Laboratory Animals and approved by the Animal Welfare Committee of Phoenix Bio Co., Ltd. All mice were housed individually and maintained in accordance with the Animal Ethics Committee of PhoenixBio (resolution #2214).

HBV-infected mice were randomized into four different treatment groups based on body weight, blood human albumin (h-Alb), and serum HBV DNA concentrations. All mice had blood h-Alb levels above 12 mg/mL and serum HBV DNA levels above 6.8 × 10^8^ copies/mL. Mice received an oral dose of 20 mg-eq/kg of HAP_R10, 100 mg-eq/kg of GS-SBA-1P, 5 mg-eq/kg of TAF, or dosing vehicle (vehicle control) once daily for 84 days. Mice were followed for an additional 28 days following cessation of treatment at day 84. Mouse serum was monitored weekly for serum HBV DNA and HBV antigens and biweekly for human (h) ALT1. Serum HBsAg and HBeAg concentrations were determined by SRL, Inc. (Tokyo, Japan) based on Chemiluminescent Enzyme Immuno Assay. HBV DNA concentration was performed using the TaqMan Fast Advanced Master Mix (Applied Biosystems, Thermo Fisher Scientific Inc.) and Applied Biosystems 7500 Real-Time PCR System (Applied Biosystems). Serum h-ALT1 concentration was determined by the Institute of Immunology Co., Ltd. (Tokyo, Japan) based on Enzyme-Linked ImmunoSorbent Assay.

### Pharmacokinetic studies

To evaluate the pharmacokinetics of HAP_R10 at steady state, uninfected uPA-SCID mice were treated orally with 20 mg/kg HAP_R10 once daily for 7 days. Blood samples on day 7 were collected at 0.5 h, 2 h, 8 h, and 24 h post dose following QD dosing of HAP_R10 for 7 days. Liver samples were collected at 24 h following the last dose of HAP_R10 to infected uPA-SCID mice. For *in vivo* efficacy study in the immunocompetent AAV-HBV mouse model, plasma samples were collected at 0, 0.5, 2, 8, and 24 h, and liver samples were collected at 24 h following the last dose of HAP_R10 from 6 mg/kg to 20 mg/kg. The concentrations of HAP_R10 in the plasma, blood, and liver were determined by liquid chromatography-tandem mass spectrometry. Pharmacokinetic parameters, including area under the plasma concentration-time curve from time 0 to 24 h (AUC_tau_), maximal concentration (C_max_), and concentration at 24 h following the last dose (C_tau_), were determined by non-compartmental analysis using Phoenix WinNonlin 6.4 (Pharsight Corporation, Princeton, NJ).

### Immunofluorescence of tissue samples

Automated singleplex immunofluorescence assay was executed on formalin-fixed, paraffin-embedded tissues for HBc (HBV core antigen; clone 366–2, rabbit IgG). Dewaxing and unmasking of antigens were performed on a Bond RX autostainer (Leica Biosystem), and subsequent assay modified for a single round of staining was performed using the Opal technology workflow (Akoya Biosciences), as described previously (39).

### RNA-Seq analysis

RNA-Seq was conducted by Q2 Expression Analysis (Durham, NC) as described previously (37). On-column DNase I treatment was performed during RNA isolation with the RNeasy Mini Kit (Qiagen), and cDNA libraries were constructed using a TruSeq Stranded mRNA Library Prep Kit (Illumina, San Diego, CA). Pair-end sequencing was conducted using Illumina HiSeq2000 with read length of 50 nucleotides. On average, approximately 30 million reads were generated per sample. Sequencing reads were aligned to the human, mouse, and HBV genomes according to the STAR method (41).

Differential gene expression (DGE) was performed using DESeq2 (v1.33.4) (42). Differentially expressed genes were defined as Benjamini Hochberg adjusted *P* values < 0.05 and |Log2FoldChange| > 1. GSEA was performed using fgsea package (v1.12.0) with -nperm = 10,000 parameter. Ranks used were derived from DGE analysis with DESeq2. The gene sets were Hallmark Pathways (v7.0) and Canonical Pathways (v7.0), including KEGG, BIOCARTA, REACTOME, and WIKIPATHWAYS.

### siRNA knockdown

p62 siRNA knockdown studies were performed in HBV-infected cells PHH plated either in a 12-well plate with glass cover-slips or 96-well plate format and infected with HBV as described. Three days post-infection, transfection mixtures were prepared by diluting siRNAs (Thermo Fisher) in Opti-MEM (GIBCO) and combining with RNAiMax transfection reagent (Thermo Fisher 13778075). siRNA was purchased from Thermo Fisher: p62 (SQSTM1) s16961, 4392420. Transfection complexes were formed at room temperature, and 20 nM siRNA was added to the plated cells. Treatment with HAP_R10 at 20× EC50 (300 nM) or DMSO began 3 days post-transfection. Media with compound or DMSO were refreshed every 3–4 days for 14 days. In the 12-well plates, immunostaining and confocal analysis of cells were performed for p62 and HBc as described. In the 96-well plates, supernatants and cells were harvested for HBV marker analysis and for cell viability by the Alamar Blue assay (Thermo Fisher DAL1025). siRNA knockdowns were validated by qRT-PCR analysis of the targeted mRNA. All experiments were performed with at least two biological replicates and several technical repeats.

### Western blot analysis

Cultured cells (HepAD38, HepG2-NTCP, and PHH) and liver tissue (from AAV-HBV and uPA-SCID) were washed with PBS and lysed with Biomasher II tube (Kimble, 749625–0030) in RIPA lysis buffer (ThermoFisher, 89900) with Halt protease inhibitor cocktail (ThermoFisher, 78430). Clarified cell and tissue extracts were boiled in 1× NuPage LDS sample buffer (Invitrogen; NP0007) and 1× NuPage Sample Reducing Agent (Invitrogen; NP0009). Equal protein extracts were loaded onto a 4%–12% Bis-Tris gel and transferred to nitrocellulose membranes using a standard iBot 2 system (Invitrogen). Membranes were blocked in Odyssey blocking buffer (LI-COR, 927–60001), and proteins of interest were analyzed using primary antibodies as follows: anti-HBc (1 µg/mL, in-house), anti-HBsAg (1 µg/mL, Fitzgerald,20-HR20), and β-actin (1:1,000; LI-COR; 926–42210). Secondary antibodies were used IRDye 680-conjugated or 800-conjugated Goat anti-mouse or goat anti-rabbit antibodies (1:10,000; LI-COR Biosciences). Signal intensities were quantified using Image Studio Lite quantification software.

### Immunofluorescence analysis of human samples

A subset of baseline samples from the 102/103 study (*n* = 43; 21 eAg− and 22 eAg+) was analyzed as previously described (39) with slight modifications. Briefly, stained images were used to first identify individual cells (using Na + K + -ATPase and nuclei marker) followed by using HBc marker to quantify average HBc intensity per cell using Visiopharm application (Visiopharm, Denmark).

### Data analysis

Antiviral activity or cytotoxicity of each test compound was determined from, extracellular HBV DNA, HBeAg, HBsAg, and alamarBlue data by comparing compound-treated PHH to DMSO-treated PHH to generate a percent of DMSO control value (% DMSO control). The % DMSO control was calculated by the following equation: % DMSO Control = 100× (XC/XD) where XC is the signal from the compound-treated PHH, and XD is the signal from the DMSO-treated PHH. The percentage of DMSO control for HBV DNA, HBeAg, HBsAg, and alamarBlue was plotted vs the log of each compound concentration in GraphPad Prism (version 6; GraphPad Software, La Jolla, CA) to generate dose-response curves. EC_50_ values were defined as the test compound concentration that caused a 50% decrease in HBV DNA, HBeAg, or HBsAg. CC_50_ values were defined as the test compound concentration that caused a 50% decrease in alamarBlue. The top of the dose-response curves was constrained to 100 and fitted using the nonlinear regression equation “log(agonist) vs response—Variable slope (four parameters)” in GraphPad Prism to determine EC_50_ or CC_50_ values. Error bars represent SD. Statistical analyses were performed by two-way analysis of variance (ANOVA) and Pearson’s correlation tests.

## Data Availability

RNA-Seq data generated in this study were deposited in the Gene Expression Omnibus (http://www.ncbi.nlm.nih.gov/geo) with accession number GSE260966.

## SUPPLEMENTAL MATERIAL

Supplemental figures aac.00420-24-s0001.pdf Figures S1 to S8.

Supplemental text aac.00420-24-s0002.docx Supplemental methods.

Supplemental tables aac.00420-24-s0003.docx Tables S1 to S6.

ASM does not own the copyrights to Supplemental Material that may be linked to, or accessed through, an article. The authors have granted ASM a non-exclusive, world-wide license to publish the Supplemental Material files. Please contact the corresponding author directly for reuse.
