# Supplementary material for: Selective depletion of HBV-infected hepatocytes by class A capsid assembly modulators requires high levels of intrahepatic HBV core protein
Source: Antimicrob Agents Chemother. 2024 May 23;68(7):e00420-24. doi: 10.1128/aac.00420-24 (PMC11232385; doi:10.1128/aac.00420-24)
Supplement: Supplemental figures — Figures S1 to S8. [file aac.00420-24-s0001.pdf]

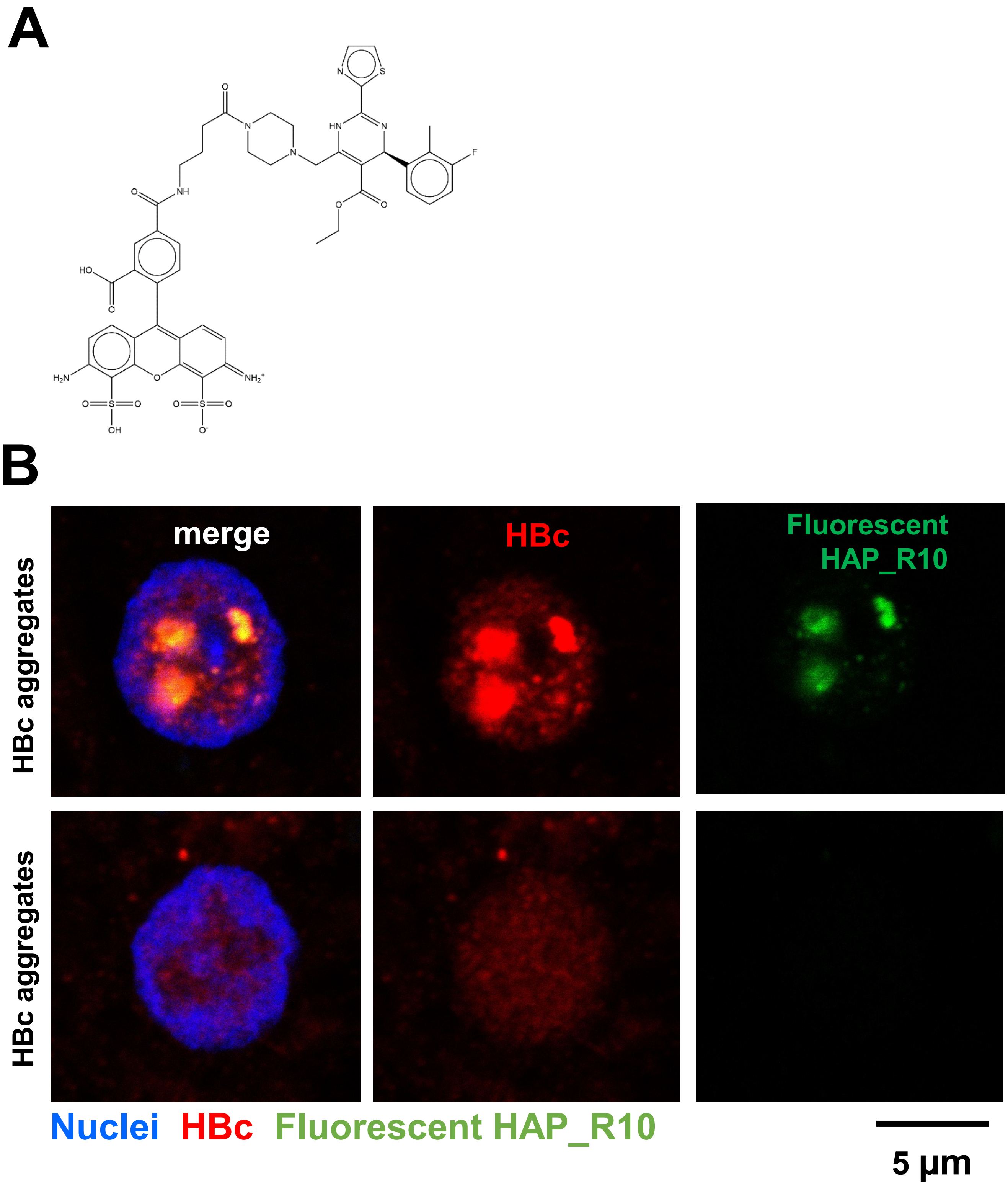

Supplemental Figure 1. Fluorescently labelled HAP_R10 is present at the site of HBc aggregation. A. Chemical structure of the fluorescently labelled HAP_R10. B. Representative confocal images of HBV-infected PHH treated with 300 nM fluorescently labeled HAP_R10 (green) for 10 days and stained for HBc (red) and nuclei (blue).

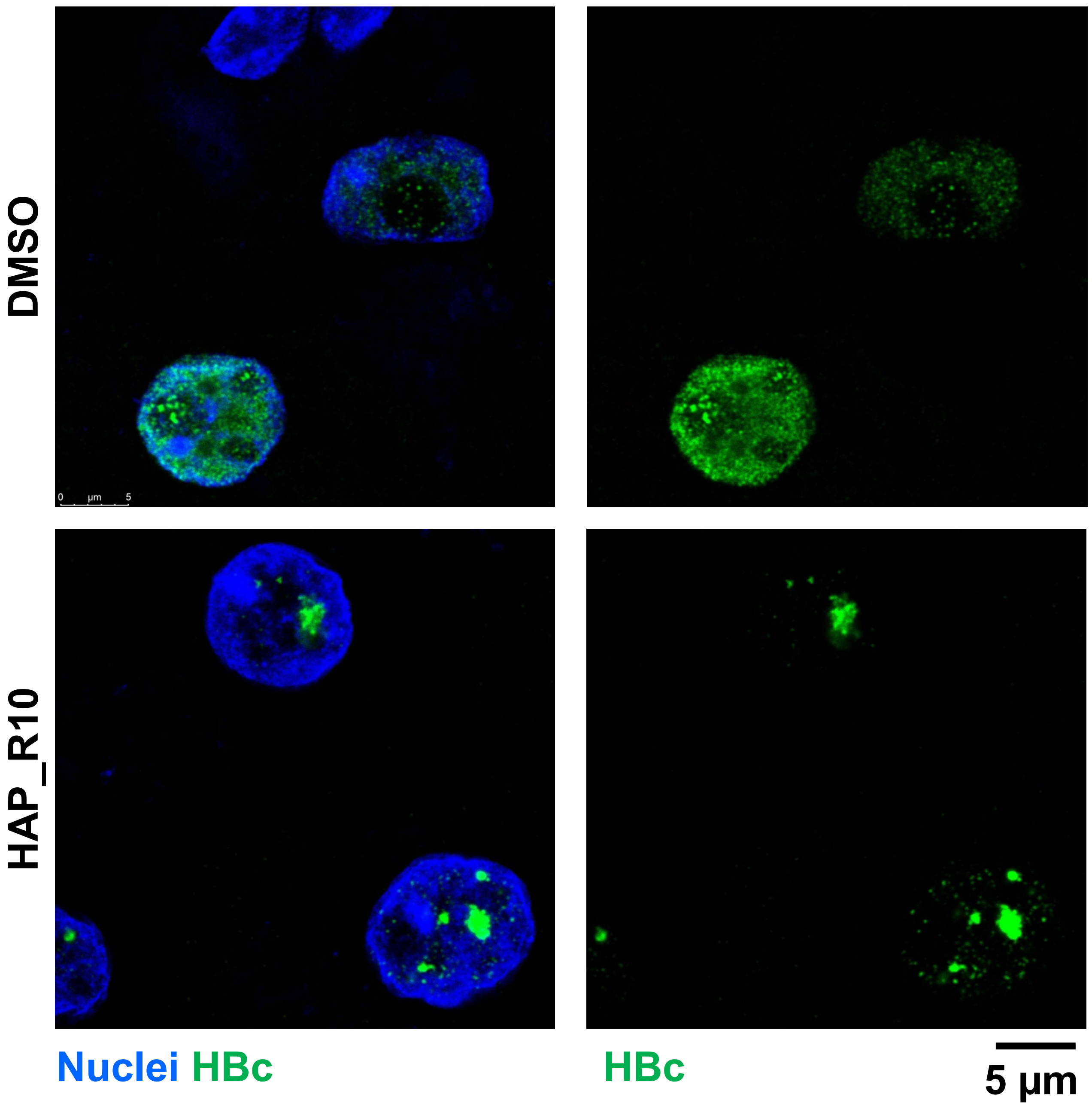

Supplemental Figure 2. Expression of HBc only leads to formation of HBc aggregates with HAP_R10 treatment in PHH. Representative confocal images of PHH transfected with HBc mRNA and treated with 20x EC_{50} (300 nM) HAP_R10 for 9 days. Cells were stained for HBc (green) and nuclei (blue).

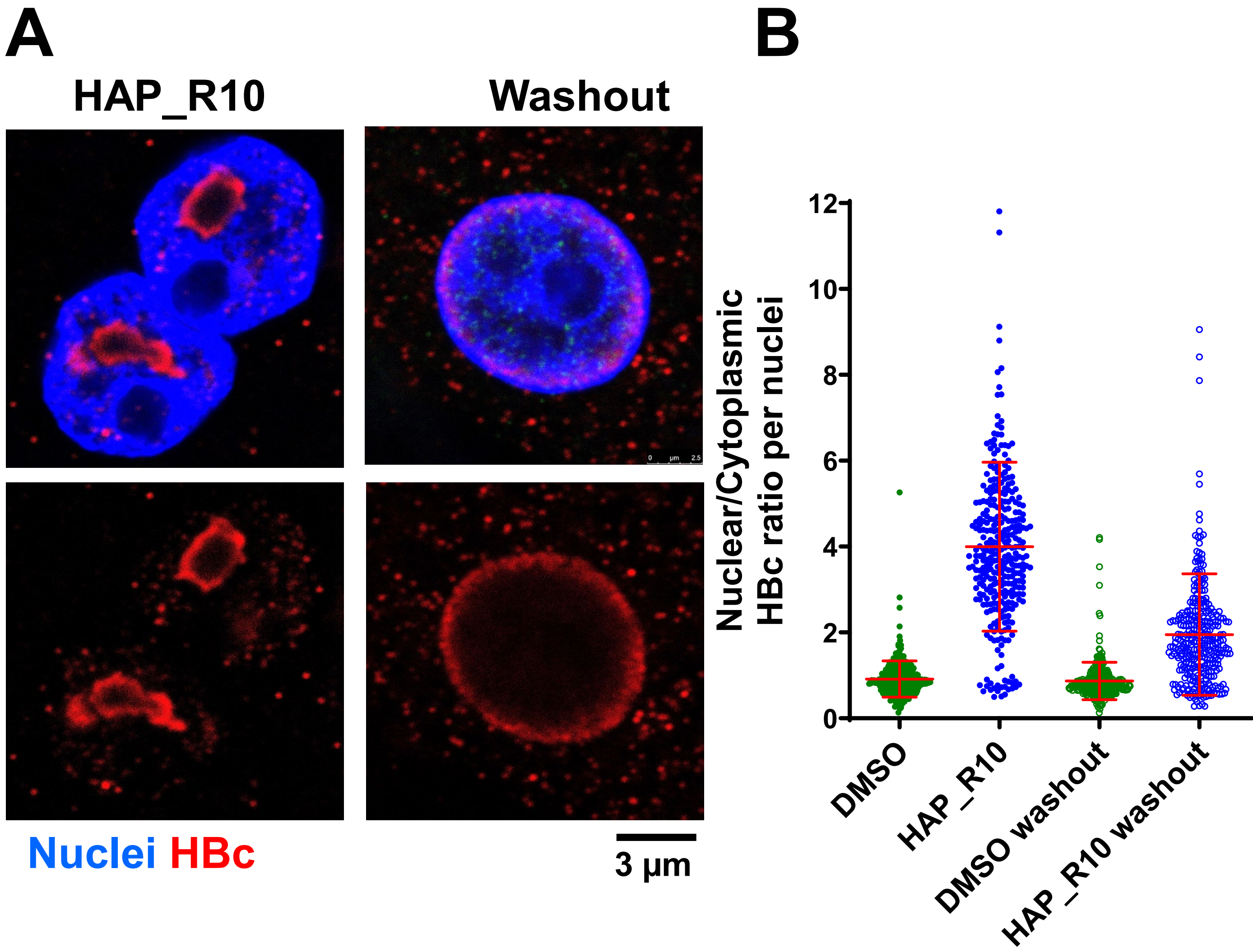

Supplemental Figure 3. HBc aggregates disappear following HAP_R10 washout. (A-B) HBV-infected PHH were treated with 300 nM HAP_R10 for 3 days following 3-day compound washout or compound treatment. (A) Representative confocal images of PHH stained for HBc (red) and nuclei (blue). (B) Nuclear/ cytoplasmic HBc ratio with HAP_R10 treatment with and without compound washout. Data is shown as HBc signal per individual nuclei with mean ± SD (n=320-365 nuclei per variant).

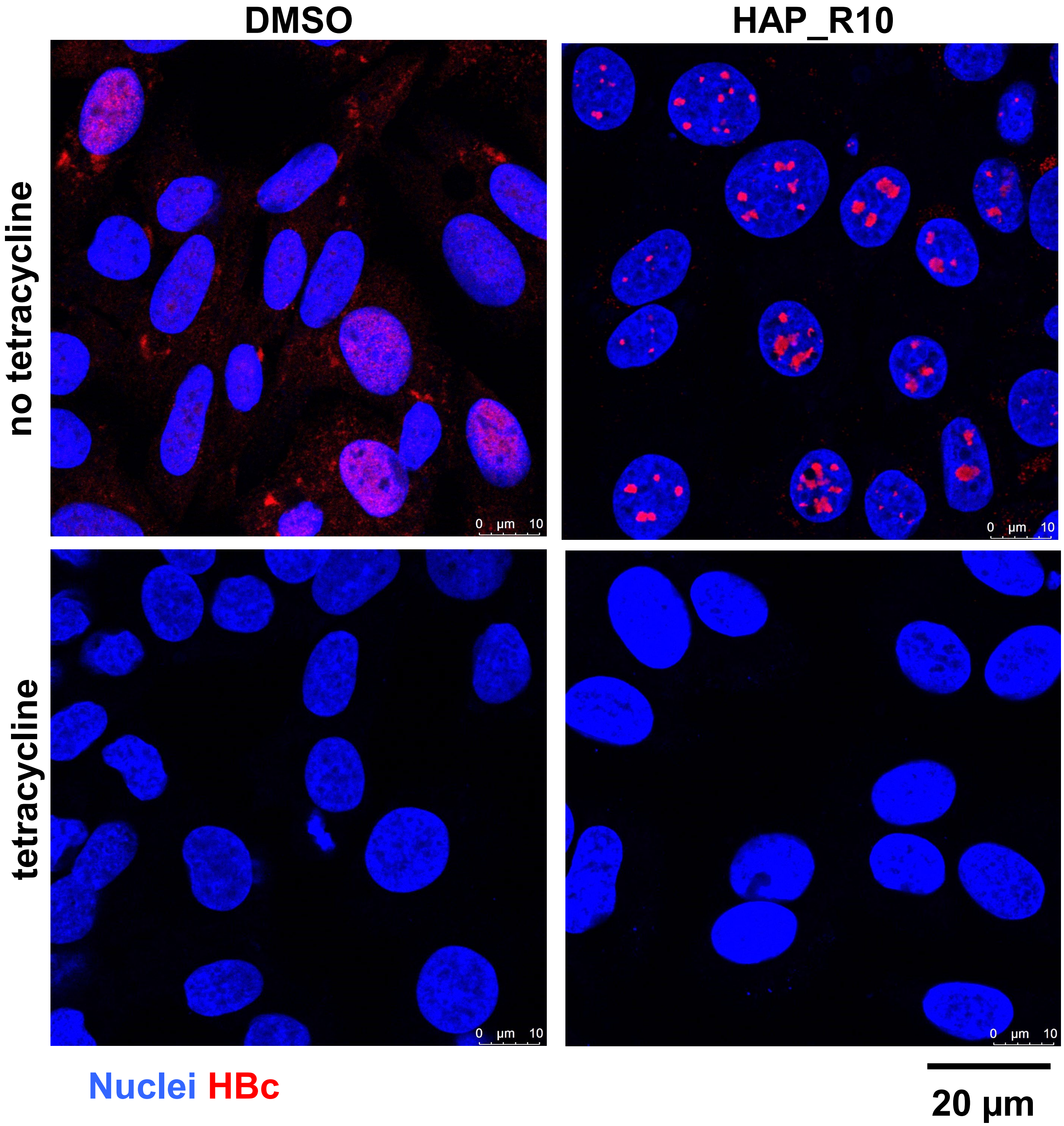

Supplemental Figure 4. HBc aggregates form in HepG2 AD38 cells with HAP_R10 treatment. Representative confocal images of HepG2 AD38 cells treated with 20x EC_{50} (300nM) HAP_R10 or DMSO for 6 days with and without tetracycline. Cells were stained for HBc (red) and nuclei (blue).

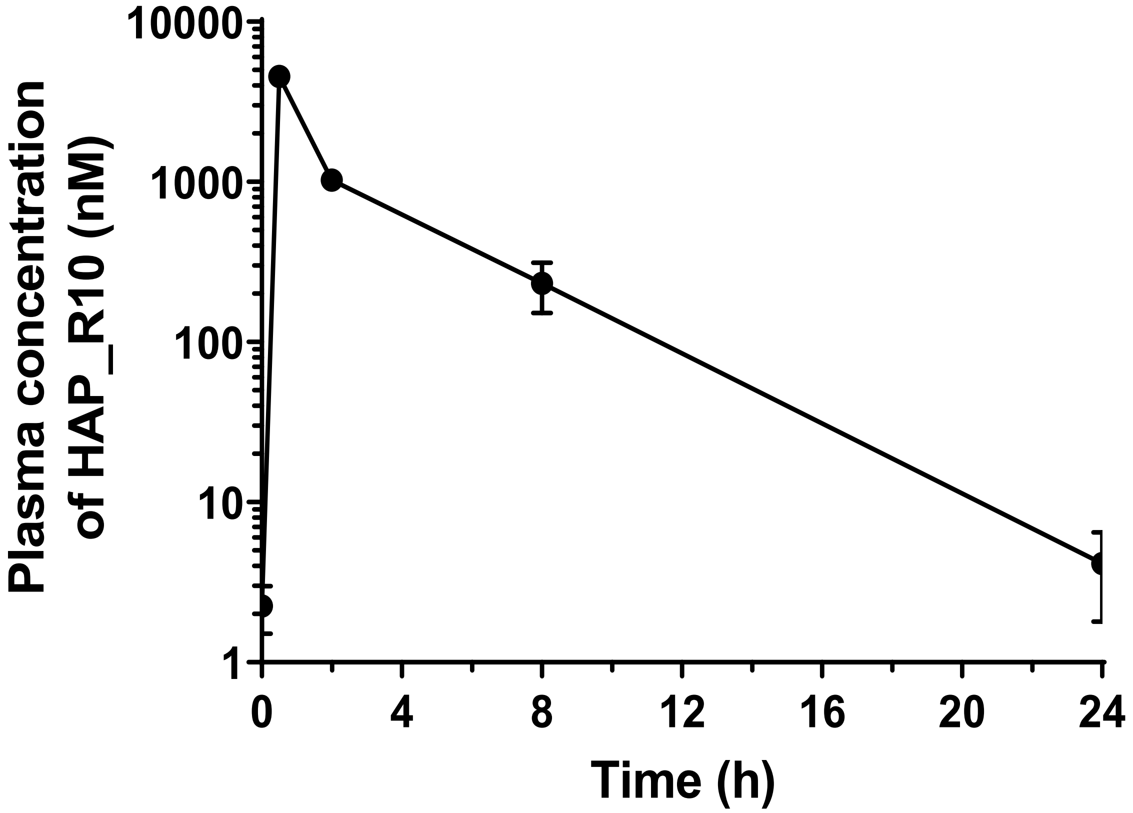

Supplemental Figure 5. Pharmacokinetic studies in AAV-HBV mouse model. Concentration of HAP_R10 in mouse plasma samples collected at 0, 0.5, 2, 8 and 24 h following the last dose of HAP_R10 at 20 mg/kg.

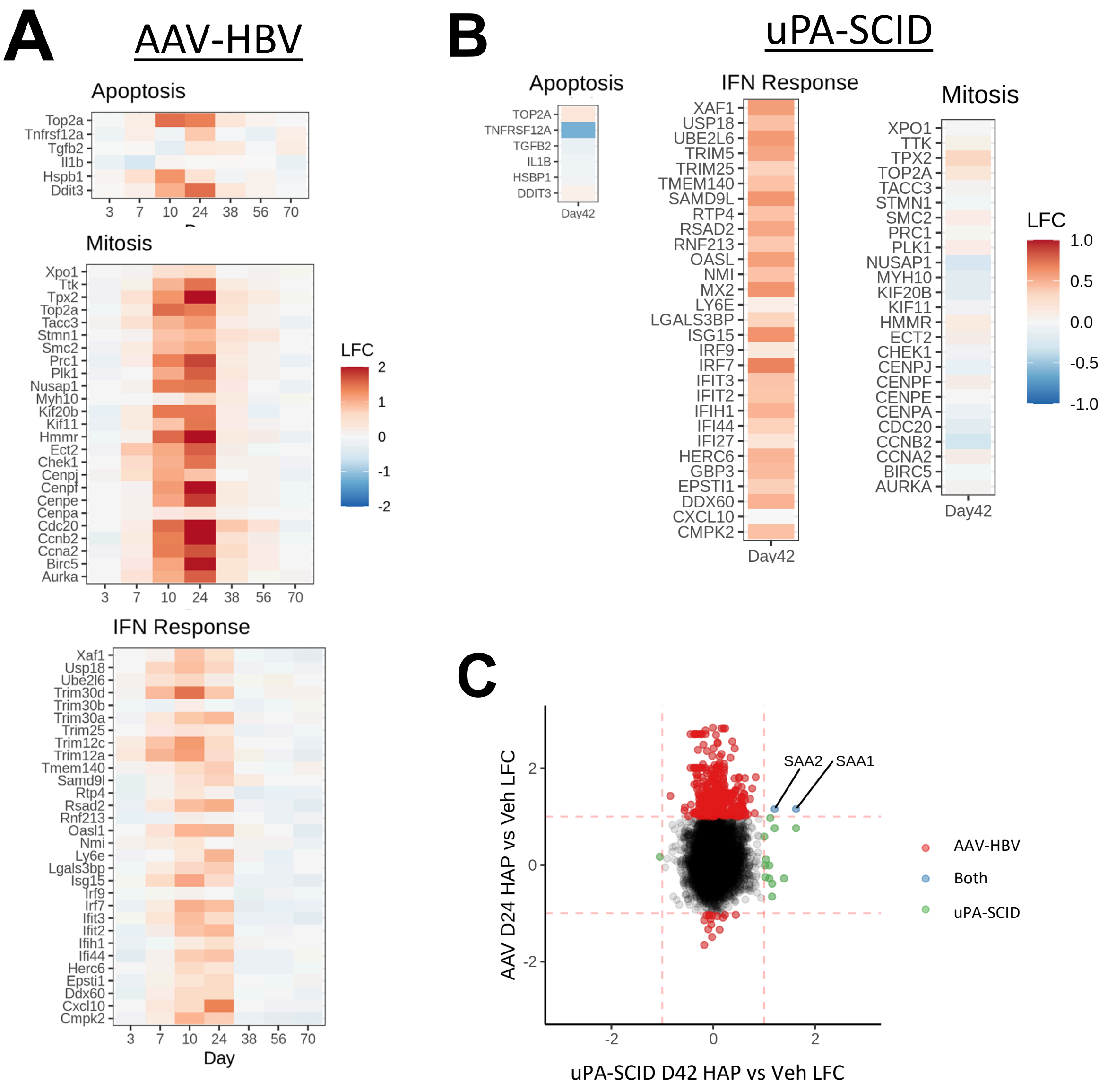

Supplemental Figure 6. Gene expression changes in animal studies related to apoptosis, mitosis and interferon pathways. (A) AAV animal study heatmaps show $\log_2$ fold-changes (LFC) relative to infected time-matched vehicle control. (B) Animal study with humanized liver mouse (uPA-SCID). LFC are relative to infected vehicle control. (C) LFC vs LFC between AAV day 24 HAP vs vehicle and PXB day 42 HAP vs vehicle. Highlighted genes are called significant in both datasets. Significance defines as FDR<0.05 and |LFC|>1.

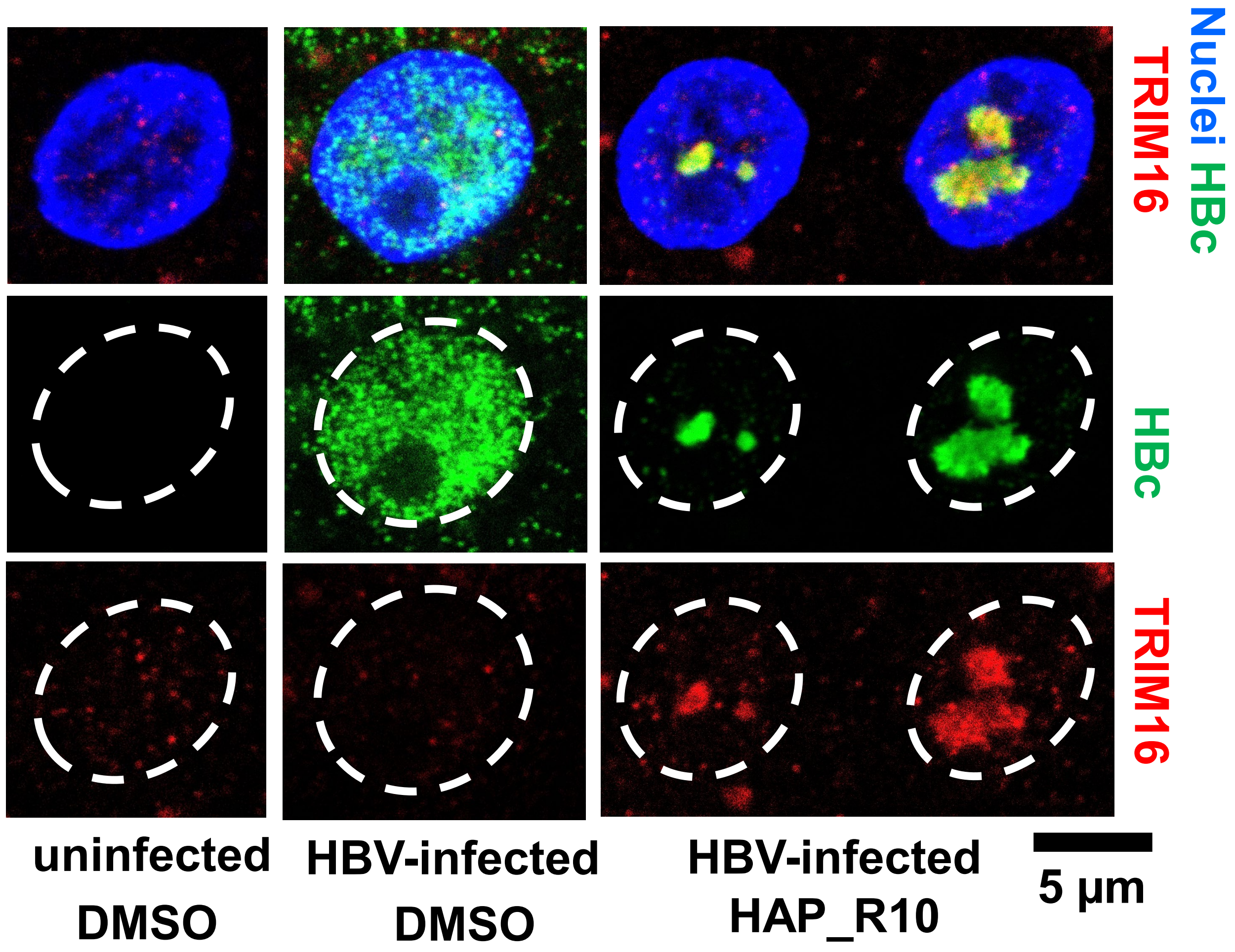

Supplemental Figure 7. Colocalization analysis of HBc aggregates and TRIM16 in HAP_R10 treated HBV-infected PHH. Representative confocal images of uninfected PHH and HBV-infected PHH, which were treated with 20X EC_{50} (300 nM) HAP_R10 for 10 days. PHH were stained for HBc (green), TRIM16 (red) and nuclei (blue). Boundaries of nuclei are outlined with dashed white line based on DAPI signal (blue).

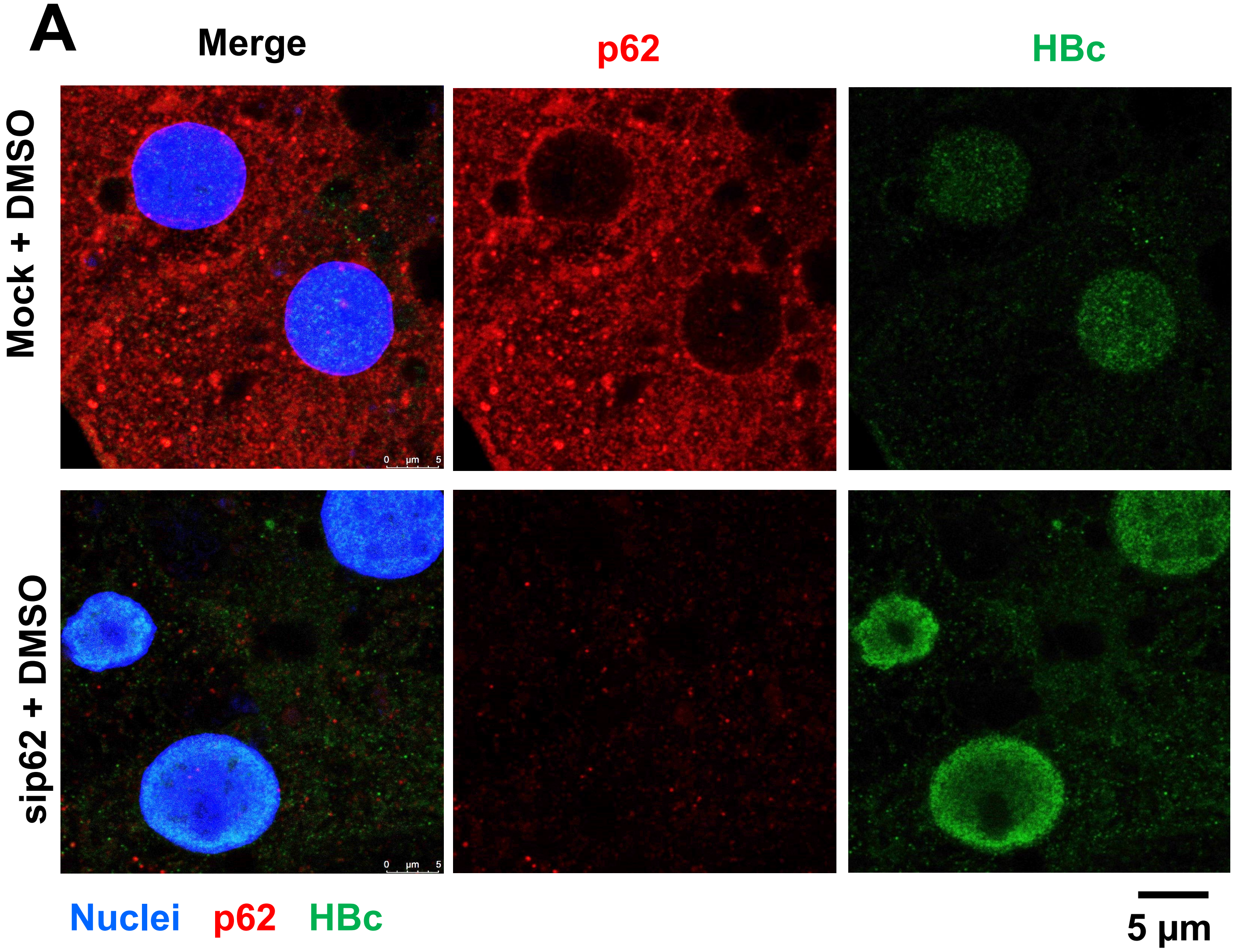

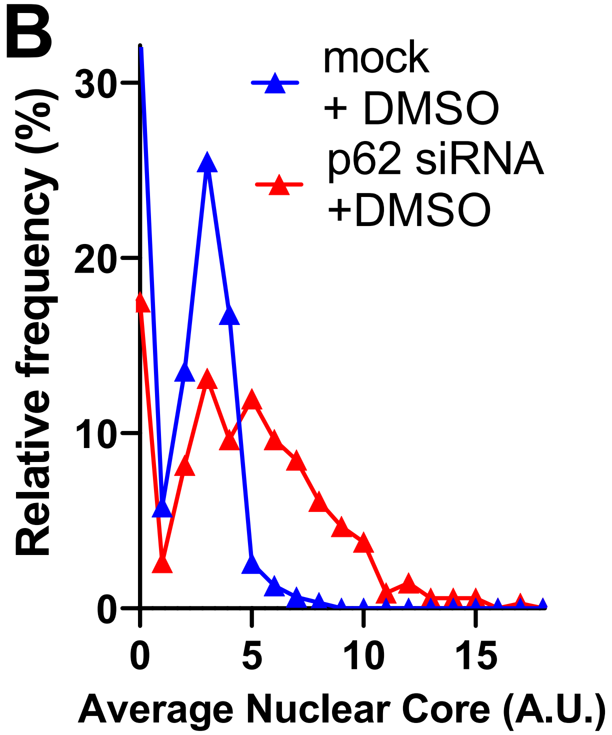

Supplemental Figure 8. HBc localization analysis with p62 siRNA knockdown in HBV-infected PHH in the absence of CAMs. (A) Representative confocal images of HBV-infected PHH in the presence of siRNA-mediated knockdown of p62. PHH were stained for p62 (red), HBc (green) and nuclei (blue). (B) Frequency histogram of the average fluorescence signal of HBc per nucleus.
