# Supplementary material for: Selective depletion of HBV-infected hepatocytes by class A capsid assembly modulators requires high levels of intrahepatic HBV core protein
Source: Antimicrob Agents Chemother. 2024 May 23;68(7):e00420-24. doi: 10.1128/aac.00420-24 (PMC11232385; doi:10.1128/aac.00420-24)
Supplement: Supplemental text — Supplemental methods. [file aac.00420-24-s0002.docx]

**Supplemental Materials and Methods**

**Quantification of Intracellular Viral RNA**

Intracellular HBV viral RNA (vRNA) was isolated from PHH using the RNeasy 96 kit (Qiagen, 74182) following the manufacturer’s recommended protocol. Quantification of vRNA by qRT-PCR was performed as previously described in (1).

**Fluorescent HAP_R10**

PHH were plated in 12-well plates with glass cover-slips and infected with HBV as described. Treatment with fluorescently labeled HAP_R10 at 300 nM (20x EC_50_) or DMSO began 3 days post-infection. Media with compound or DMSO was refreshed every 3 to 4 days for additional 10 days. Immunostaining and confocal analysis of cells for HBc was performed as described in the main text. All experiments were performed with at least two biological replicates.

**HBc mRNA transfection in PHH**

PHH were plated in 12-well plates with glass cover-slips as described. One day later, transfection mixtures were prepared with 0.005ug/ul HBV core mRNA (HBV Genotype D AD38) and Opti-MEM (GIBCO) and combined with Trans-IT mRNA Transfection Kit (Mirrus MIR2250), following the manufacturer’s protocol. Two days later, cells were re-transfected with HBc mRNA and treated with HAP_R10 at 300 nM (20x EC_50_) or DMSO. Re-transfection and compound or DMSO treatment was repeated every 2 to 3 days. After 7 days of HAP_R10 or DMSO treatment, immunostaining and confocal analysis of cells for HBc were conducted. All experiments were performed with at least two biological replicates and several technical repeats.

**HBc aggregates in HepG2 AD38 cells**

HepG2 AD38 cells were plated in 12-well plates with glass coverslips and grown in media with tetracycline as described. To induce HBV replication, growth media was replaced with assay media without tetracycline the next day. After three days, treatment with HAP_R10 at 300 nM (20x EC_50_) or DMSO began and assay media with compound or DMSO was refreshed every 3 days. After 6 days of HAP_R10 and DMSO treatment, immunostaining and confocal analysis of cells for HBc was performed as described. All experiments were performed with at least two biological replicates.

**Washout studies**

PHH were plated in 12-well plates with glass coverslips and infected with HBV as described. Treatment with HAP_R10 at 300 nM (20x EC_50_) or DMSO began 3 days post-infection, and media with compound or DMSO was refreshed every 3 days. On day 6 post-infection, cells were washed 3 times and were either replenished with media only or media with compounds. On day 9 post-infection, immunostaining and confocal analysis of cells was performed for HBc as described. All experiments were performed with at least two biological replicates.

**TRIM16 co-localization**

PHH were plated in 12-well plates with glass coverslips and infected with HBV as described. Treatment with HAP_R10 at 300 nM (20x EC_50_) or DMSO began 3 days post-infection. Media with compound or DMSO was refreshed every 3 to 4 days for an additional 10 days. At the end of the treatment, cells were fixed in paraformaldehyde and subjected to immunostaining. All experiments were performed with at least two biological replicates.

**Long-term cytotoxicity study in HepG2 AD38 cells**

HepG2 AD38 cells were plated in 12-well plates and grown in media with tetracycline. To induce HBV replication, growth media was replaced with assay media without tetracycline the next day. After three days, treatment with HAP_R10 at 300 nM (20x EC_50_) or DMSO began and assay media with compound or DMSO was refreshed every 3-4 days. In parallel, cells were treated with compound or DMSO in media with tetracycline. After 7, 14, 21, and 28 days of compound treatment, cells were trypsinized, counted, and re-plated. The study duration spanned 34 days. Cell viability was determined by dividing the number of cells with HAP_R10 treatment by the DMSO control. All conditions were performed with at least two biological replicates at each time point.

**Long-term cytotoxicity study in HBV-infected HepG2-NTCP and PHH**

HepG2-NTCP cells and PHH were infected with HBV for 19 and 38 days, respectively as described. After three days, treatment with HAP_R10 at 300 nM (20x EC_50_) or DMSO began and assay media with compound or DMSO was refreshed every 3-4 days. Extracellular HBV DNA, and HBeAg / HBsAg were measured by qPCR and an electrochemiluminescence assay (MSD). Cell toxicity was measured with AlamarBlue (ThermoFisher, DAL1025) or CellTiter-Glo (Promega, G7572).

1. Burdette D, Hyrina A, Song Z, Beran RK, Cheung T, Gilmore S, Kobayashi T, Li L, Liu Y, Niedziela-Majka A, Medley J, Mehra U, Morganelli P, Novikov N, Niu C, Tam D, Tang J, Wang J, Yue Q, Fletcher SP, Holdorf MM, Delaney WEt, Feierbach B, Lazerwith S. 2023. Characterization of a Novel Capsid Assembly Modulator for the Treatment of Chronic Hepatitis B Virus Infection. Antimicrob Agents Chemother 67:e0134822.
