# Supplementary material for: Selective depletion of HBV-infected hepatocytes by class A capsid assembly modulators requires high levels of intrahepatic HBV core protein
Source: Antimicrob Agents Chemother. 2024 May 23;68(7):e00420-24. doi: 10.1128/aac.00420-24 (PMC11232385; doi:10.1128/aac.00420-24)
Supplement: Supplemental tables — Tables S1 to S6. [file aac.00420-24-s0003.docx]

**Supplemental Tables**

**Supplemental Table 1.** Antiviral activity of HAP_R10 in PHH treated at the time of HBV infection.

| **Assay** |  |
| --- | --- |
| HBV DNA EC_50_ (µM) | 0.026 |
| Intracellular HBV RNA EC_50_ (µM) | 2.1 |
| HBsAg EC_50_ (µM) | 1.5 |
| HBeAg EC_50_ (µM) | 1.4 |
| Cytotoxicity CC_50_ (µM) | >4.5 |

**Supplemental Table 2.** Effect of HAP_R10 on cell toxicity in different HBV cell culture systems. HAP_R10 treatment was performed at 20xEC_50_.

| **Cell system** | **Days of treatment** | **HBV DNA reduction, % (±SD)** | **HBeAg reduction, % (±SD)** | **HBsAg reduction, % (±SD)** | **Cell Toxicity** |
| --- | --- | --- | --- | --- | --- |
| HepAD38 | 34 | 93 (±0.5) | 72 (±3) | 36 (±4) | No |
| HBV-infected HepG2-NTCP | 19 | 100 (±0.00) | 77 (±5) | 50 (±11) | No |
| HBV-infected PHH | 38 | 99 (±0.05) | 71 (±7) | 70 (±3) | No |

**Supplemental Table 3.** Pharmacokinetic parameters from AAV-HBV mouse model. Mouse plasma samples were collected at 0, 0.5, 2, 8 and 24 hours and the liver samples were collected at 24 hours following the last dose of HAP_R10 at 20 mg/kg. Data is shown as mean ± SD, n=4.

| **Plasma PK Parameters** | **HAP_R10 (± SD), n=4** |
| --- | --- |
| **AUC_tau_ (µM•h)** | 11.0 (± 1.9) |
| **C_max_ (µM)** | 4.56 (± 0.70) |
| **C_tau_ (µM)** | 0.0041 (± 0.0047) |
| **Liver C_tau_ (µM)** | 0.53 (± 0.051) |

**Supplemental Table 4.** Gene expression changes related to apoptosis, mitosis and interferon pathways in the HBV-infected livers from chimeric humanized mice with HAP_R10 compared to vehicle control at day 42 of treatment. FDR, false discovery rate.

| **Group** | **Gene** | **log2FoldChange** | **FDR** |
| --- | --- | --- | --- |
| IFN | CMPK2 | 0.44 | 0.089 |
| IFN | CXCL10 | -0.00099 | 0.99 |
| IFN | DDX60 | 0.51 | 0.049 |
| IFN | EPSTI1 | 0.38 | 0.17 |
| IFN | GBP3 | 0.47 | 0.0066 |
| IFN | HERC6 | 0.49 | 0.060 |
| IFN | IFI27 | 0.22 | 0.37 |
| IFN | IFI44 | 0.37 | 0.20 |
| IFN | IFIH1 | 0.50 | 0.052 |
| IFN | IFIT2 | 0.42 | 0.14 |
| IFN | IFIT3 | 0.43 | 0.12 |
| IFN | IRF7 | 0.69 | 0.0038 |
| IFN | IRF9 | 0.18 | 0.30 |
| IFN | ISG15 | 0.63 | 0.016 |
| IFN | LGALS3BP | 0.36 | 0.11 |
| IFN | LY6E | 0.12 | 0.48 |
| IFN | MX2 | 0.63 | 0.0075 |
| IFN | NMI | 0.44 | 0.047 |
| IFN | OASL | 0.58 | 0.022 |
| IFN | RNF213 | 0.41 | 0.030 |
| IFN | RSAD2 | 0.55 | 0.016 |
| IFN | RTP4 | 0.44 | 0.12 |
| IFN | SAMD9L | 0.62 | 0.0044 |
| IFN | TMEM140 | 0.44 | 0.040 |
| IFN | TRIM25 | 0.37 | 0.052 |
| IFN | TRIM5 | 0.55 | 0.015 |
| IFN | UBE2L6 | 0.60 | 0.016 |
| IFN | USP18 | 0.44 | 0.093 |
| IFN | XAF1 | 0.59 | 0.016 |
| apoptosis | DDIT3 | 0.088 | 0.70 |
| apoptosis | HSBP1 | -0.074 | 0.55 |
| apoptosis | IL1B | -0.083 | NA |
| apoptosis | TGFB2 | -0.13 | 0.69 |
| apoptosis | TNFRSF12A | -0.62 | 0.0026 |
| apoptosis | TOP2A | 0.21 | 0.56 |
| mitosis | AURKA | 0.046 | 0.91 |
| mitosis | BIRC5 | -0.037 | 0.93 |
| mitosis | CCNA2 | 0.091 | 0.84 |
| mitosis | CCNB2 | -0.30 | 0.38 |
| mitosis | CDC20 | -0.19 | 0.61 |
| mitosis | CENPA | -0.082 | NA |
| mitosis | CENPE | -0.0067 | 0.99 |
| mitosis | CENPF | 0.095 | 0.83 |
| mitosis | CENPJ | -0.12 | 0.67 |
| mitosis | CHEK1 | -0.063 | 0.87 |
| mitosis | ECT2 | 0.101 | 0.79 |
| mitosis | HMMR | 0.128 | 0.75 |
| mitosis | KIF11 | -0.055 | 0.90 |
| mitosis | KIF20B | -0.19 | 0.52 |
| mitosis | MYH10 | -0.18 | 0.32 |
| mitosis | NUSAP1 | -0.27 | 0.31 |
| mitosis | PLK1 | 0.11 | 0.77 |
| mitosis | PRC1 | 0.028 | 0.95 |
| mitosis | SMC2 | 0.12 | 0.68 |
| mitosis | STMN1 | -0.028 | 0.94 |
| mitosis | TACC3 | 0.048 | 0.88 |
| mitosis | TPX2 | 0.34 | 0.25 |
| mitosis | TTK | 0.088 | 0.84 |
| mitosis | XPO1 | 0.0081 | 0.97 |

**Supplemental Table 5.** Changes in p62/sqstm1 expression in the livers from AAV-HBV mice treated with HAP_R10 compared to vehicle control.

| **Gene** | **Day** | **log2FoldChange** | **FDR** |
| --- | --- | --- | --- |
| Sqstm1 | 3 | 0.17 | 0.074 |
| Sqstm1 | 7 | 0.23 | 0.036 |
| Sqstm1 | 10 | 0.27 | 1.12E-06 |
| Sqstm1 | 24 | 0.45 | 7.61E-04 |
| Sqstm1 | 38 | 0.088 | 0.69 |
| Sqstm1 | 56 | 0.030 | 0.85 |
| Sqstm1 | 70 | 0.162 | 0.28 |

**Supplemental Table 6.** CHB patients’ characteristics.

| **CHB patient** | **Serum HBeAg** | **Serum HBV DNA (log10 IU/mL)** | **Serum HBsAg (log 10 IU/ml)** | **ALT (U/L)** | **Serum HBcrAg (Log10 U/mL)** |
| --- | --- | --- | --- | --- | --- |
| 1 | + | 7.53 | 4.31 | 191 | 8.2 |
| 2 | + | 8.43 | 4.55 | 144 | 7.9 |
| 3 | + | 8.52 | 4.52 | 126 | 8.3 |
| 4 | + | 8.31 | 4.66 | 76 | 8.5 |
| 5 | + | 8.06 | 3.55 | 77 | 8.1 |
| 6 | + | 8.26 | 4.74 | 670 | 7.9 |
| 7 | + | 8.45 | 4.89 | 210 | 7.25 |
| 8 | + | 9.93 | 5.16 | 118 | 8.9 |
| 9 | + | 8.94 | 5.13 | 98 | 7.75 |
| 10 | + | 7.96 | 4.78 | 120 | 6.3 |
| 11 | + | 6.77 | 4.62 | NA | 6.1 |
| 12 | + | 7.82 | 4.54 | 77 | 7.5 |
| 13 | + | 7.95 | 4.10 | 179 | 7.6 |
| 14 | + | 8.05 | 4.82 | 134 | 7.6 |
| 15 | + | 7.97 | 3.32 | 68 | 8.2 |
| 16 | + | 8.97 | 5.34 | 105 | 9.0 |
| 17 | + | 7.65 | 4.74 | 125 | 6.8 |
| 18 | + | 8.73 | 5.07 | 123 | 8.6 |
| 19 | + | 6.57 | 3.80 | 184 | QNS |
| 20 | + | 7.91 | 4.63 | 80 | 8.4 |
| 21 | + | 8.44 | 5.32 | 107 | 7.35 |
| 22 | + | 8.44 | 5.32 | 107 | 7.35 |
| 1 | - | 5.76 | 3.15 | 119 | 3.6 |
| 2 | - | 7.26 | 4.07 | 92 | 6.3 |
| 3 | - | 6.24 | 3.58 | 106 | 4.6 |
| 4 | - | 7.93 | 4.29 | 98 | 7.1 |
| 5 | - | 6.84 | 4.21 | 71 | 5.1 |
| 6 | - | 6.53 | 1.44 | 291 | QNS |
| 7 | - | 5.78 | 4.03 | 424 | 5.4 |
| 8 | - | 7.56 | 3.07 | 280 | 3.8 |
| 9 | - | 7.56 | 3.81 | 204 | 6.6 |
| 10 | - | 7.13 | 3.86 | 85 | 5.8 |
| 11 | - | 8.10 | 4.40 | 97 | 7.1 |
| 12 | - | 6.91 | 3.61 | 139 | 5.6 |
| 13 | - | 7.68 | 4.73 | 884 | 6.9 |
| 14 | - | 7.59 | 4.05 | 114 | NA |
| 15 | - | NA | 4.07 | 81 | NA |
| 16 | - | 5.96 | 3.72 | 234 | 4.2 |
| 17 | - | 7.68 | 4.73 | 884 | 6.9 |
| 18 | - | 7.27 | 4.33 | 162 | NA |
| 19 | - | NA | 4.07 | 81 | NA |
| 20 | - | 6.12 | NA | 320 | 5.0 |
| 21 | - | 7.39 | 3.41 | 225 | NA |

*NA, not available; QNS, quantity not sufficient
